# Extension of Sous Vide Preservation of Wild Turkey ( *Meleagris gallopavo* L.) Meat With *Rosmarinus officinalis* Essential Oil: A Study on Chemical Composition and Antibacterial Effectiveness Against *Listeria monocytogenes* and Other Population of Isolated Microbiota

**DOI:** 10.1111/1758-2229.70299

**Published:** 2026-05-29

**Authors:** Miroslava Kačániová, Guiguo Zhang, Suzana Popovic, Alessandro Bianchi, Zhaojun Ban, Li Li, Natália Čmiková, Joel Horacio Elizondo‐Luevano, Anis Ben Hsouna, Rania Ben Saad, Peter Haščík, Stefania Garzoli

**Affiliations:** ^1^ Institute of Horticulture, Faculty of Horticulture and Landscape Engineering Slovak University of Agriculture Nitra Slovakia; ^2^ School of Medical & Health Sciences VIZJA University Warszawa Poland; ^3^ Department of Animal Nutrition Shandong Agricultural University Taian City China; ^4^ China‐South Korea International Joint Laboratory of Functional Polysaccharides in Shandong Province Taian City China; ^5^ Centre for Molecular Medicine and Stem Cell Research, Faculty of Medical Sciences University of Kragujevac Kragujevac Serbia; ^6^ Department of Agriculture, Food and Environment University of Pisa Pisa Italy; ^7^ School of Biological and Chemical Engineering Zhejiang University of Science and Technology, Zhejiang Provincial Key Laboratory of Chemical and Biological Processing Technology of Farm Products, Zhejiang Provincial Collaborative Innovation Center of Agricultural Biological Resources Biochemical Manufacturing Hangzhou China; ^8^ Key Laboratory for Agro‐Products Postharvest Handling of Ministry of Agriculture and Rural Affairs, College of Biosystems Engineering and Food Science Zhejiang University Hangzhou China; ^9^ Department of Chemistry, Faculty of Biological Sciences Universidad Autónoma de Nuevo León San Nicolás de los Garza Nuevo León Mexico; ^10^ Laboratory of Biotechnology and Plant Improvement, Centre of Biotechnology of Sfax Sfax Tunisia; ^11^ Department of Environmental Sciences and Nutrition, Higher Institute of Applied Sciences and Technology of Mahdia University of Monastir Monastir Tunisia; ^12^ Institute of Food Technology, Faculty of Biotechnology and Food Sciences Slovak University of Agriculture Nitra Slovakia; ^13^ Department of Chemistry and Technologies of Drug Sapienza University Rome Italy

**Keywords:** antimicrobial activity in vitro, chemical composition, microbiological analysis, microbiota identification, rosemary essential oil, wild turkey meat testing in vivo

## Abstract

In our study, we focused on the chemical composition of *Rosmarinus officinalis* essential oil (ROEO), its antimicrobial effects. Drawing from existing research, we further investigated the antimicrobial properties of ROEO under in vivo conditions. Specifically, we examined its effects on sous vide meat from wild turkeys and its activity against *Listeria monocytogenes* . The dominant compound identified in ROEO was 1,8‐cineole, which made up 48.5% of the oil. During our experiments, we assessed the total viable count, coliform numbers, and the presence of *L. monocytogenes* on sous vide wild turkey meat. Furthermore, we analysed the abundance of various bacterial species in each of the tested groups. Our findings suggest that the ROEO‐treated groups exhibited a lower microbial load compared to the control groups or those treated solely with pathogenic microorganisms. Our microbial counts revealed that heat treatment had a noticeable effect on the microbial populations. ROEO demonstrated significant antimicrobial properties, as our results indicated its positive impact across all experimental groups. Additionally, mass spectrometry results highlighted differences in the microbiota composition following the application of ROEO and the inoculation of *L. monocytogenes* . The primary bacterial species isolated from the sous vide wild turkey meat were *Citrobacter freundii* and *Pantoea agglomerans* .

## Graphical abstract

Schematic illustration of sous vide preparation of wild turkey meat and microbiological analysis of the meat.

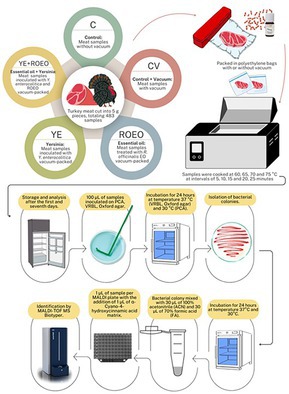

## 1 Introduction

Milk and meat products are essential components of the everyday diet, yet they are particularly susceptible to microbial contamination. These foods are also frequent carriers of various foodborne pathogens. Common pathogens found in these products include *Escherichia coli* O157:H7, *Salmonella*, *Listeria monocytogenes* , *Yersinia enterocolitica* , *Campylobacter jejuni* , *Clostridium perfringens* , *Staphylococcus aureus* , and *Toxoplasma gondii* (Keba et al. 2020). To extend shelf life and reduce the risk of foodborne illness, the meat industry has incorporated several antimicrobial treatments, including both synthetic preservatives and antibiotics. However, the growing concern about antibiotic resistance and the adverse effects of synthetic chemicals has led to increased interest in natural antimicrobial agents as alternatives (Wu‐Wu et al. 2023). Natural substances such as bacteriocins, lactoferrin, lysozyme, spices, essential oils, and plant extracts have been explored for their antimicrobial potential in meat products. Among these, recent studies highlighted the effectiveness of spices as preservatives, demonstrating their ability to inhibit microbial growth (Chauhan and Rao 2024; Duda‐Chodak et al. 2023; Santiesteban‐López et al. 2022).

Essential oils represent an promising natural alternative in food preservation, and their use is becoming a key area of focus in food safety (Rout et al. 2022). Rosemary ( *Rosmarinus officinalis* L.) a member of the Lamiaceae family, is a well‐known aromatic shrub with a distinctive scent described as pine‐like. Native to Mediterranean regions, including Spain, Morocco, Tunisia, France, and Italy, rosemary has been used for centuries to enhance culinary flavour (Flamini et al. 2002). Today, it is also recognised for its therapeutic properties, owing to the diverse bioactive compounds found in its essential oils. These compounds exhibit a range of beneficial effects, including antimicrobial, antispasmodic, carminative, hepatoprotective, antiviral, and anticancer properties (Bozin et al. 2007). Rosemary can be used both fresh and dried, though the latter is often more practical for storage and transportation, as dried herbs retain their quality over time (Díaz‐Maroto et al. 2007). Understanding how drying methods impact the essential oil content and properties of rosemary is crucial, as different drying techniques can affect its chemical composition (Szumny et al. 2010). In this study, we deliberately selected *Listeria monocytogenes* as a model pathogen due to its psychrotolerance and relevance to ready‐to‐eat meats; accordingly, we do not extrapolate our findings to other pathogens.

Over the past few years, substantial research has focused on the volatile compounds in rosemary essential oil. The oil is primarily composed of monoterpenes and their derivatives, which make up 95%–98% of the total composition, with sesquiterpenes accounting for the remaining 2%–5% (Angioni et al. 2004; Díaz‐Maroto et al. 2007). Key volatiles identified in rosemary oil include camphor, 1,8‐cineole, borneol, verbenone, α‐pinene, and camphene (Becer et al. 2023; Calín‐Sánchez et al. 2011; Machado et al. 2024; Rafya et al. 2024; Rathore et al. 2022; Sakar et al. 2023).

The increasing consumer demand for minimally processed, ready‐to‐eat foods, combined with the globalisation of the food supply chain, has heightened the risk of foodborne infections (Martins and Leal Germano 2011). One of the most concerning pathogens in ready‐to‐eat foods is *Listeria monocytogenes* , as it is capable of surviving and growing at low temperatures. Essential oils from *Rosmarinus officinalis* have been shown to possess antibacterial properties, helping to reduce spoilage and pathogenic bacteria in food products (Oliveira et al. 2010; Souza et al. 2007). However, research has indicated that higher concentrations of rosemary essential oil are often required to effectively inhibit bacterial growth, which may exceed levels that are organoleptically acceptable (Naveena et al. 2006; Souza et al. 2009). Among the major components of rosemary essential oil, α‐pinene has been reported as a major contributor to significantly inhibiting *L. monocytogenes* (Schneider et al. 2023), while other ROEO constituents may also contribute to the overall effect. Recent studies also employ kinetic assays to monitor time‐dependent progression of antimicrobial activity against *L. monocytogenes* (Cacciatore et al. 2022; Coimbra et al. 2022; Rasooli et al. 2006), and these tests are crucial for understanding the mechanisms underlying the antimicrobial effects of specific essential oils (Guo et al. 2021).

While research on the microbiology of poultry, particularly chicken, is well‐established (Höll et al. 2016; Kunert‐Filho et al. 2022; Oakley et al. 2013; Saenz‐García et al. 2020), fresh turkey meat has received less attention (Jaber et al. 2017; Saucier et al. 2000). It is imperative to better understand the microbiota and populations of major bacterial communities commonly found in raw turkey meat, including Enterobacteriaceae, enterococci, staphylococci, and *Pseudomonas* spp. The primary source of microbial contamination in slaughterhouse carcasses is faecal matter, making *Enterobacteriaceae* and *E. coli* valuable indicators of hygiene (Dias Costa et al. 2023). *Enterococci*, typically present in animal intestines, can contaminate turkey carcasses if sanitation practices are inadequate during processing (Bortolaia et al. 2016). *Listeria monocytogenes* is a widespread pathogen in the meat industry, often originating from environmental contamination rather than the raw materials themselves (Autio et al. 2000; Barbuti and Parolari 2002; Colak et al. 2007). The use of slicing machines in food processing has been identified as a common route for the spread of *L. monocytogenes* in ready‐to‐eat (RTE) deli meats (Lin et al. 2006). Sous vide cooking offers a significant advantage in preserving food quality while maintaining its sensory attributes, providing precise temperature control for consistent texture and doneness (Baldwin 2021). However, low‐temperature cooking can present food safety risks if pasteurisation guidelines are not strictly followed, as it may allow pathogens to survive, potentially leading to product degradation and reduced shelf life (Baldwin 2021).

The objective of this study was to evaluate the antimicrobial effects of *R. officinalis* essential oil (ROEO) against *Listeria monocytogenes* under both in vitro and in vivo conditions. Additionally, we investigated the growth kinetics of *L. monocytogenes* with and without the addition of ROEO, as well as the effects of sous vide cooking conditions on wild turkey meat. This study also provides valuable insights into the microbiota of wild turkeys (*Meleagris gallopavo L*.), identifying microorganisms that are not commonly reported in previous research. The findings contribute to a better understanding of the microbial ecology of wild turkey meat and the impact of various treatments on the identified species.

## 2 Material and Methods

### 2.1 Rosemary Essential Oil

The antimicrobial properties of rosemary essential oil (ROEO), derived from *Rosmarinus officinalis* L., were evaluated in this study. The essential oil was sourced from Hanus Ltd. (Nitra, Slovakia) and produced via steam distillation of the flowering twigs from a plant originating in Tunisia. The oil was stored at 4°C prior to analysis.

### 2.2 GC/MS Analysis of *R. officinalis* Essential Oil

An Agilent 6890 N/5975B gas chromatograph‐mass spectrometer (Agilent Technologies, Santa Clara, CA) equipped with an HP‐5MS fused silica column (5% phenylmethyl polysiloxane, 30 m, 0.25 mm i.d., film thickness 0.25 μm, Agilent Technologies), was used to analyse the volatiles in rosemary essential oil. The chromatographic conditions were as follows: injector and transfer line temperatures were set at 250°C and 280°C, respectively; oven temperature programmed at 50°C with an increase of 3°C/min to 75°C (isothermal for 4 min), 5°C/min from 75°C to 120°C (isothermal for 2 min) and then 5°C/min to 290°C; carrier gas, helium at 1 mL/min; injection of 1 μL (10% hexane solution of the ROEO); split ratio 1:40.8; MS source temperature 230°C; MS quadruple temperature 150°C; mass scan range, 35–500 amu at 70 eV. Chromatographic and mass spectrometric data acquisition for the injected essential oil sample started after a solvent delay of 3.20 min, with a total run time of 57.33 min. To obtain the retention times and mass spectra of individual *n*‐alkanes (C7–C35 mixture), the solvent delay time was set at 2.30 min, with the fact that the acquisition continued until the GC oven maintained a final temperature of 290°C for 8.00 min (resulting total run time, 65.33 min). The volatile constituents in the ROEO were identified by comparing the recorded mass spectra with those in the Wiley7 and NIST libraries, and by correlating the collected MS data and experimentally calculated Kovats retention indices with literature data (Adams 2007). Compounds with concentrations higher than 0.1% were semi‐quantified using the MSD ChemStation integrator (Agilent Technologies, Santa Clara, CA, USA) using their respective GC peak regions.

### 2.3 Bacterial Strains Used in This Study

The bacterial strain *Listeria monocytogenes* CCM 4699 was obtained from the Czech Collection of Microorganisms (Brno, Czech Republic) for this study. The bacterial inoculum was cultivated on Mueller‐Hinton agar (MHA, Oxoid, Basingstoke, UK) at 37°C for 24 h. The optical density was adjusted to the 0.5 McFarland standard (1.5 × 10^8^ CFU/mL). The inoculum was mixed with 100 μL of wild turkey breast samples to ensure even distribution of the pathogen. Following a 3‐min gentle shaking at room temperature, the samples were inoculated with *L. monocytogenes* . All experiments in this study were conducted exclusively with *Listeria monocytogenes* ; no other bacterial species were evaluated.

### 2.4 Disc Diffusion Method

The antimicrobial efficacy of ROEO was evaluated using the disc diffusion method (DDM). Bacterial cultures were grown in Mueller‐Hinton Broth (MHB, Oxoid, Basingstoke, UK) at 37°C for 24 h. The bacterial suspension was adjusted to the 0.5 McFarland standard (1.5 × 10^8^ CFU/mL) and spread onto MHA plates. Sterile 6‐mm paper discs were saturated with 10 μL of ROEO and placed on the agar surface. After incubating the plates at 37°C for 24 h, the inhibition zones (IZ) surrounding each disc were measured in triplicate.

### 2.5 Minimal Inhibition Concentration (MIC)

The MIC of ROEO was determined using a 96‐well microplate method. Bacteria were cultured in MHB (Oxoid, Basingstoke, UK) at 37°C for 24 h, followed by adjusting the optical density to match the 0.5 McFarland standard. ROEO was diluted in MHB, resulting in concentrations ranging from 10 mg/mL to 0.00488 mg/mL, which were added to each well. The microplate was incubated at 37°C for 24 h, with absorbance measured at 570 nm using a Glomax spectrophotometer (Promega Inc., Madison, WI, USA). The MIC values were calculated as the lowest concentrations of ROEO that inhibited 50% (MIC_50_) and 90% (MIC_90_) of bacterial growth. Each trial was performed in triplicate.

### 2.6 Kinetic Growth of *L. monocytogenes* With and Without ROEO

The influence of ROEO and temperature on the growth kinetics of *Listeria monocytogenes* was assessed using a personal bioreactor (RTS‐1, Biosan, Riga, Latvia), which monitored optical density (OD) at 850 nm. After culturing the strain on MHA at 37°C for 24 h, a single colony was transferred to 100 mL MHB and mixed thoroughly. The culture was distributed into two 23 mL samples in sealed tubes with perforated caps, and placed in the bioreactor at 37°C until the culture reached the exponential growth phase (OD_850_ = 1). At this point, 10 mg/mL of ROEO was added to one tube, while the other served as a control. The bioreactor gradually increased the temperature to 50°C, and OD readings were taken at 5, 10, 15, and 20‐min intervals. The experiment was repeated at 55°C, 60°C, and 65°C to evaluate the effects of ROEO and heat stress on bacterial growth.

### 2.7 Shelf‐Life Extension of Sous‐Vide Wild Turkey Meat

#### 2.7.1 Preparation of Sous‐Vide Wild Turkey Meat

Wild turkeys used in this study were raised on a small farm in Slovakia. During the first three weeks, birds received a complete turkey feed; thereafter the diet consisted of feed plus grazing (mainly grasses), and in the finishing period wheat, barley, and maize with grazing. Birds hatched in May and were raised until November. Wild turkey meat was transported under refrigeration to the microbiology laboratory. The meat was cut with a sterile knife into 25 g portions and assigned to experimental groups (total 483 samples). On day 0, three raw samples were analysed; on days 1 and 7, 240 samples each (control and treated) were analysed across time/temperature combinations.

ROEO (food‐grade rosemary essential oil) was prepared at 1% (v/w) in sunflower oil and applied directly to the surface of samples assigned to ROEO treatments. Samples for sous‐vide processing were vacuum‐packed in polyethylene high‐barrier bags. Controls without vacuum were packed in polyethylene bags without evacuation.

For inoculated groups, each sample received 100 μL of a 0.5 McFarland suspension of *Listeria monocytogenes* CCM 4699 (1.5 × 10^8^ CFU/mL) distributed evenly over the surface, followed by gentle mixing (1 min) to ensure uniform contact before sealing. This corresponded to 1.5 × 10^7^ CFU per sample.

Thermal processing was carried out in a water bath (CASO SV1000, Arnsberg, Germany) at 50°C, 55°C, 60°C, and 65°C for 5, 10, 15, or 20 min. After heating, samples were cooled and stored at 4°C until analysis.

Experimental groups:

- Control (C): packed without vacuum; heated as above
- Control + vacuum (CV): vacuum‐packed; heated as above
- Essential oil: treated with 1% ROEO, vacuum‐packed; heated as above
-
*L. monocytogenes* : inoculated with *L. monocytogenes* , vacuum‐packed; heated as above.
- Essential oil + LM: treated with 1% ROEO and inoculated with *L. monocytogenes* , vacuum‐packed; heated as above.

#### 2.7.2 Microbiological Evaluation of Wild Turkey Meat

Microbiological analyses were performed on day 1 and day 7 of storage at 4°C. For enumeration (Kačániová, Garzoli, et al. 2024), 25 g of meat were aseptically transferred to sterile stomacher bags with 225 mL peptone water (1:10 dilution) and homogenised in a stomacher (15 min). Appropriate decimal dilutions were surface‐plated (100 μL) as follows:

- Coliform bacteria: Violet Red Bile Lactose agar (VRBL; Oxoid, Basingstoke, UK), 37°C/24–48 h.
- Total viable count (TVC): Plate Count Agar (PCA; Oxoid), 30°C/48–72 h.
-
*Listeria monocytogenes* : Oxford agar (OA; Oxoid) supplemented with Oxford Listeria Selective Supplement SR0140, 37°C/24–48 h.

Counts were expressed as log CFU/g.

#### 2.7.3 Bacterial Identification by MALDI‐TOF MS Biotyper

Representative colonies were identified using the MALDI‐TOF MS Biotyper (Bruker Daltonics, Bremen, Germany) with the manufacturer's reference library. The α‐cyano‐4‐hydroxycinnamic acid (HCCA) matrix was prepared in 50% acetonitrile with 2.5% trifluoroacetic acid. For extraction, biomass from isolated colonies was suspended in distilled water, mixed with ethanol, pelleted (10,000 g × 2 min), air‐dried, and resuspended in 70% formic acid followed by acetonitrile (1:1, 30 μL + 30 μL). One microlitre of extract and 1 μL of HCCA matrix were spotted on the target plate and air‐dried. Identification scores were interpreted as follows: ≥ 2.000 reliable species‐level identification; 1.700–1.999 probable genus‐level identification; < 1.700 unreliable (Kačániová, Garzoli, et al. 2024).

### 2.8 Statistical Analysis

Data are expressed as the mean ± the standard deviation (SD), and each analysis was performed in triplicate. One‐way ANOVA was conducted, and the Tukey's HSD test was carried out at a significantly *p* ≤ 0.05 level (CoStat version 6.451, CoHort Software, Pacific Grove, CA, USA). Finally, JMP Pro 17.0 software (SAS Institute, Cary, NC, USA) was used for the graphic processing.

## 3 Results and Discussion

### 3.1 Chemical Composition of ROEO

Based on the results of the GC/MS analysis shown in Table 1, the tested sample of rosemary essential oil contains twenty‐five compounds (99.9% of total). Monoterpenes, represented by twenty‐two identified compounds (96.6% of total oil), are the most abundant class of volatiles. The sesquiterpene class, comprising three identified compounds, was present in small amounts (3.3% of total oil). Monoterpene ether 1,8‐cineole was the most abundant compound (48.5%) and can therefore be classified as a cineoliferous (high 1,8‐cineole content) chemotype (Pauli and Schilcher 2010; Rafya et al. 2024). Also, significant concentrations of the monoterpene hydrocarbons *α*‐pinene (11.3%) and *β*‐pinene (8.5%), along with the monoterpene ketone camphor (10.9%), were observed.

**TABLE 1:** Chemical composition and classes of identified compounds in *Rosmarinus officinalis* essential oil.

No	Compound^a^	%^b^	KI^c^	
			(lit.)	(calc.)
1	tricyclene	tr^d^	926	924
2	*α*‐thujene	0.3	930	927
3	*α*‐pinene	11.3	939	935
4	camphene	4.8	951	954
5	sabinene	tr	975	972
6	*β*‐pinene	8.5	979	978
7	*β*‐myrcene	1.2	990	988
8	*α*‐phellandrene	tr	1002	1006
9	*δ*‐3‐carene	tr	1011	1008
10	*α*‐terpinene	tr	1017	1018
11	*ο*‐cymene	1.5	1026	1027
12	limonene	2.3	1029	1032
13	1,8‐cineole	48.5	1031	1038
14	*γ*‐terpinene	0.6	1059	1063
15	*p*‐mentha‐3,8‐diene	tr	1072	1075
16	linalool	0.6	1096	1099
17	camphor	10.9	1146	1152
18	borneol	2.6	1169	1175
19	4‐terpineol	0.8	1177	1182
20	*α*‐terpineol	1.7	1188	1195
21	bornyl acetate	1.0	1285	1282
22	geranyl acetate	tr	1381	1377
23	(*E*)‐caryophyllene	3.3	1419	1419
24	*α*‐humulene	tr	1454	1456
25	germacrene D	tr	1481	1475
total		99.9		

Class of compounds	Number of compounds	%
monoterpenes	**22**	**96.6**
monoterpene hydrocarbons	14	30.5
monoterpene alcohols	4	5.7
monoterpene ketones	1	10.9
monoterpene ethers	1	48.5
monoterpene esters	2	1.0
sesquiterpenes	**3**	**3.3**
sesquiterpene hydrocarbons	3	3.3

^a^ Identified compounds.

^b^ Percentage composition.

^c^ Literature and calculated values of retention indices on HP‐5MS column.

^d^ tr—percentage amounts less than 0.1%.

In contrast to our oxigenated monoterpene rich ROEO sample, the main volatiles in the essential oil from the aerial parts of *R. officinalis* from Turkey were *p*‐cymene, linalool and *γ*‐terpinene (44.0%, 20.5% and 16,6%, respectively) (Özcan and Chalchat 2008). A study by (Rasooli 2008) listed piperitone (23.6%), *α*‐pinene (14.9%), limonene (14.9%) and 1,8‐cineole (7.4%) as the major compounds. On the other hand, in a sample of ROEO from Brazil, obtained by steam distillation of leaves and stems, the main compounds were two oxygenated monoterpenes 1,8‐cineole (28.5%) and camphor (27.7%), and the monoterpene hydrocarbon *α*‐pinene (21.3%) (Takayama et al. 2016). In comparison to our ROEO sample, similar percentage amounts of major compounds 1,8‐cineole (53.6%; 42.3%), *α*‐pinene (12.3%; 11.6%), and camphor (9.6%; 10.5%) were observed in two samples of *R. officinalis* essential oil from Morocco (Oualdi et al. 2023). Besides that (Diass et al. 2023) also reported significant concentrations of 1,8‐cineole and camphor (46.3% and 10.0%, respectively). One recent research focused on ROEO as alternative control of grapevine phytopathogens reported *α*‐pinene (58.4%) and 1,8‐cineole (16.3%) as major constituents of oil (Machado et al. 2024). Additionally, a study on the antioxidant and hepatoprotective activity of a cineoliferous chemotype ROEO sample from Serbia reported high concentrations of 1,8‐cineole (43.8%), camphor (12.5%), and *α*‐pinene (11.5%) (Rašković et al. 2014). Contrary, the dominant volatiles in the ROEO sample obtained from leaves of *R. officinalis* cultivated in Northern Cyprus were camphor, verbenone, *α*‐pinene and 1,8‐cineole (15.1%, 14.3%, 13.6% and 11.8%, respectively) (Becer et al. 2023). As noted above, a review of the relevant literature revealed significant variations in the chemical composition of ROEO volatiles. These differences in chemical composition could be attributed to genetic variability, the collection of plant material at different vegetative phases, the plant part used for extraction, climatic effects, local environmental factors, post‐harvest drying and storage conditions, as well as the extraction methodology (Figueiredo et al. 2008; Machado et al. 2024; Mouahid et al. 2017; Pauli and Schilcher 2010; Rafya et al. 2024; Sabbahi et al. 2023; Yosr et al. 2013).

### 3.2 Antibacterial Action

Table 2 presents the results of the in vitro evaluation of ROEO. The antibacterial effect, as measured by the DDM, resulted in an IZ of 15.67 mm. The minimum inhibitory concentrations (MIC) were determined to be 0.127 mg/mL (MIC_50_) and 0.133 mg/mL (MIC_90_). In contrast, a study by (Vidaković Knežević et al. 2023) found that EOs from various plants, including basil, rosemary, curry, sage, garden angelica, yarrow, and hyssop, did not show inhibitory effects against several *L. monocytogenes* strains, regardless of the concentration applied. In comparison, EOs from black pepper, cassia ginger, clove, lavender, lemon, and myrtle displayed a broad range of antimicrobial activity. The oils of winter sage, thyme, oregano, and cinnamon produced the largest IZ. Our results indicate that ROEO exhibited a significantly stronger antibacterial effect against *L. monocytogenes* . Previous research suggests that the antibacterial efficacy of some EOs is more pronounced in liquid forms (Ghabraie et al. 2016; Vieira et al. 2017). The broth microdilution technique, which provides greater sensitivity, is often used to quantify the antibacterial activity of EOs (Ballester‐Costa et al. 2013). Using this method, all *L. monocytogenes* strains were effectively inhibited at low concentrations by the exceptional antibacterial properties of oregano, thyme, cinnamon, and sage EOs. Specifically, thyme and cinnamon EOs exhibited MIC values between 0.12 and 0.45 μL/mL, while oregano EO displayed MIC values ranging from 0.09 to 0.72 μL/mL. MIC values for clove EO and winter savoury ranged from 0.45–1.78 μL/mL and 0.17–1.42 μL/mL, respectively, both of which were comparable (Vidaković Knežević et al. 2023). However, our study revealed that the MIC values for ROEO were higher than those reported in other studies. These findings align with the results obtained by Mazzarrino et al. (2015) and Burt (2004). However, it is worth noting that a consistent correlation between the inhibition zone sizes and MIC values was not always evident (Ballester‐Costa et al. 2013).

**TABLE 2:** ROEO antimicrobial activity against *L. monocytogenes* in vitro. Data represent averages ± SD of three tests.

Inhibition zone by disc diffusion method (mm)	ROEO	Chloramphenicol
*Listeria monocytogenes*	15.67 ± 0.58	33.67 ± 0.58

Minimal inhibition concentration (mg/mL)	MIC_50_	MIC_90_
*Listeria monocytogenes*	0.127 ± 0.07	0.133 ± 0.04

### 3.3 *Listeria monocytogenes* Kinetic Growth

The existence of naturally occurring antimicrobial substances typically reduces microbial growth or may even entirely prevent it. Defining growth‐limiting factors is crucial, particularly when the pathogen's dose–response is minimal and no microbial growth can be tolerated in food. Many studies use a fixed inoculum size without considering how changes in the inoculum might affect the onset of growth. Although rarely addressed in microbial growth kinetics research, some studies suggest that inoculum size can indeed impact microbial development (Pascual et al. 2001).

**FIGURE 1:**
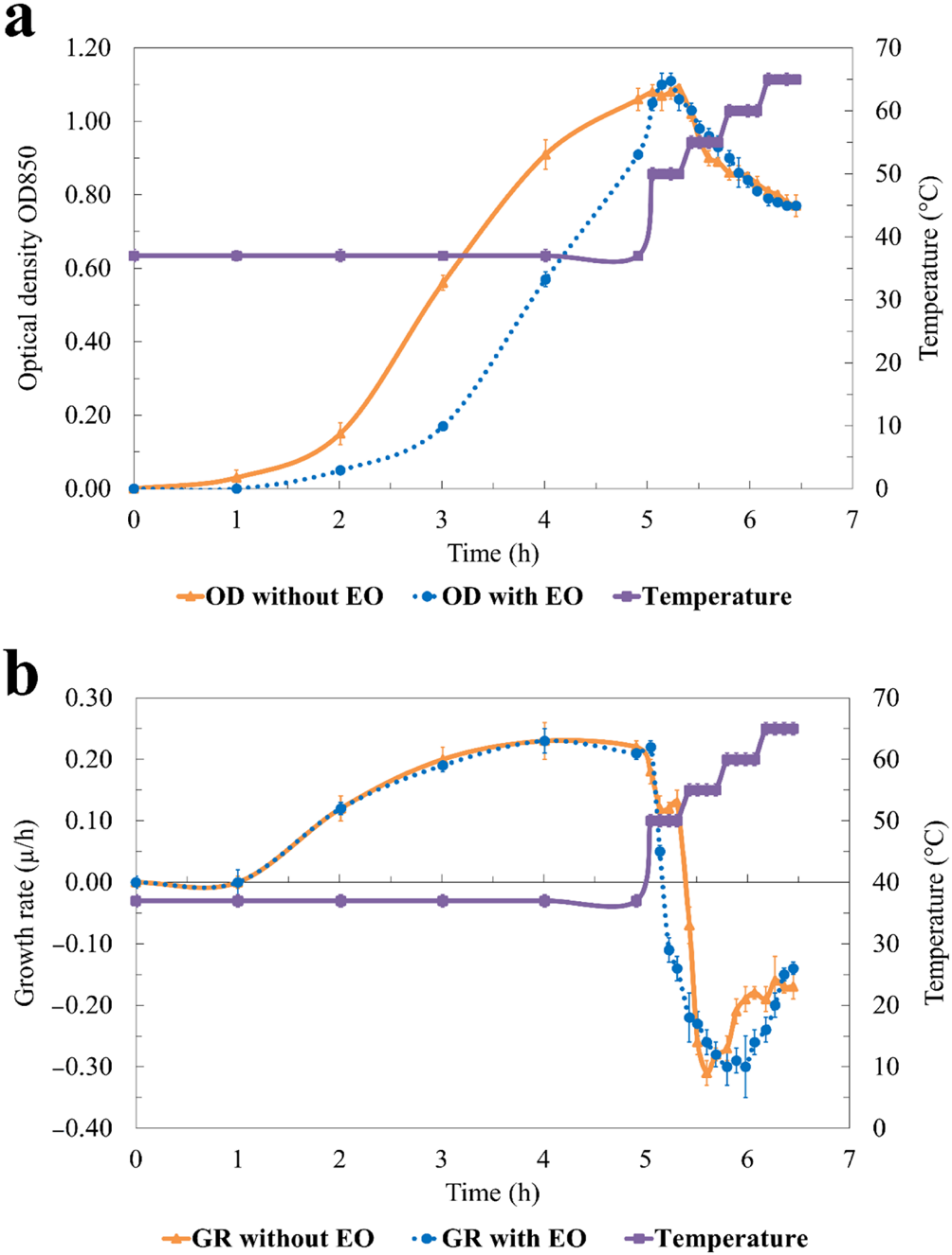
*Listeria monocytogenes* growth over time at different temperatures in ROEO‐treated (with EO) and untreated samples (without EO): (a) Optical density at 850 nm; (b) Growth rate (μ/h). Data represents the means (bars indicate the standard deviation) of 3 samples.

To evaluate the effect of rosemary EO and temperature variations on the growth of *L. monocytogenes* , optical density (OD_850_) and growth rate (GR) measurements were taken (Figure 1a,b). At each temperature (50°C–65°C), OD_850_ was recorded at 5, 10, 15, and 20 min. The EO was introduced after approximately 5 h of incubation, when the culture reached the logarithmic phase (OD 1.08). At 37°C, both the control and EO‐treated cultures showed similar growth, with OD values of 1.1 and 1.08, respectively. However, the growth rate was slightly higher in the EO‐experimental group (0.22) compared to the control (0.18). As the temperature increased to 50°C, both groups exhibited reduced growth, though the EO treatment showed some inhibitory effects. The OD of the EO‐treated group decreased to 1.03, whereas the control remained at 1.08. The growth rate in the EO‐treated group declined to −0.14, compared to −0.11 in the untreated group, indicating a growing inhibitory effect from the EO.

At 55°C, the inhibitory effect of the EO became more pronounced. The OD in the EO‐treated group dropped to 0.96, whereas the control group maintained an OD of 1.02. The growth rate in the EO‐treated group further decreased to −0.23, while the untreated group's GR was −0.07, demonstrating the EO's more significant impact at this elevated temperature.

At 60°C, both groups continued to show reduced growth. The OD values for the EO‐treated samples and the control were 0.79 and 0.83, respectively. The growth rate in the EO‐treated group decreased substantially to −0.30, while the control group's growth rate was −0.18, further supporting the EO's sustained inhibitory effect.

At 65°C, bacterial growth in both groups was severely limited. The EO‐treated samples showed an OD of 0.76, with a growth rate of −0.14, while the control group exhibited an OD of 0.77 and a GR of −0.17, emphasising the continued inhibitory impact of rosemary EO under heat stress. This research underscores the antimicrobial activity of rosemary EO in combination with elevated temperatures, particularly its enhanced ability to inhibit *L. monocytogenes* growth at higher temperatures.

Recent studies have employed kinetic assays to track the progression of the antilisterial effect (Cacciatore et al. 2022; Coimbra et al. 2022; Rasooli et al. 2006). Kinetic profiling is essential to explore the molecular changes at the genomic and proteomic levels that accompany the antimicrobial action (Guo et al. 2021), and the antibacterial mechanisms of specific EOs can be clarified through these studies.

### 3.4 Microbiological Quality of Sous Vide Wild Turkey Meat

The microbial levels in wild game meat production, particularly during the initial stages, lack standardisation, which likely explains the significant variability observed. With strict hygiene procedures, it is possible to achieve microbial counts around 3–4 log CFU/cm^2^, although some studies have documented counts exceeding 8 log CFU/cm^2^ (Paulsen 2011). Many studies have highlighted the presence of harmful bacteria in the digestive tract or on the surface of wild game, but there is limited research addressing pathogen levels in living animals and how this contributes to contamination during processing. To market wild game meat similarly to farmed animal meat, it would be reasonable to establish comparable bacterial limits as part of performance standards. It has been suggested that improving primary production practices is crucial for enhancing both the safety and shelf life of wild game meat (Paulsen 2011). In our investigation of sous vide wild turkey meat, we observed higher microbial counts than those reported for venison meat in previous studies (Kačániová, Čmiková, et al. 2024; Kačániová, Garzoli, et al. 2024). Furthermore, various studies have indicated that EOs can impact total viable counts, coliform levels, and microbiota composition, even in sous vide turkey meat, which also exhibited increased microbial levels.

We monitored the microbiological quality of wild turkey meat from day 0 (baseline) through day 1 and day 7. The parameters assessed were total viable count (TVC), coliform bacteria (CB), and the presence of *Listeria monocytogenes* (LM). Samples were assigned to treatments at 50°C–65°C for 5–20 min. Microbiological analyses were performed on day 0 (baseline, raw meat; *n* = 3) and on day 1 and day 7 (*n* = 240 per day across treatments). TVC was enumerated on PCA (30°C/48–72 h), coliforms on VRBL (37°C/24–48 h), and LM on OA (37°C/24–48 h). At baseline (day 0), the meat showed 2.78 log CFU/g coliforms and 3.23 log CFU/g TVC.

**FIGURE 2:**
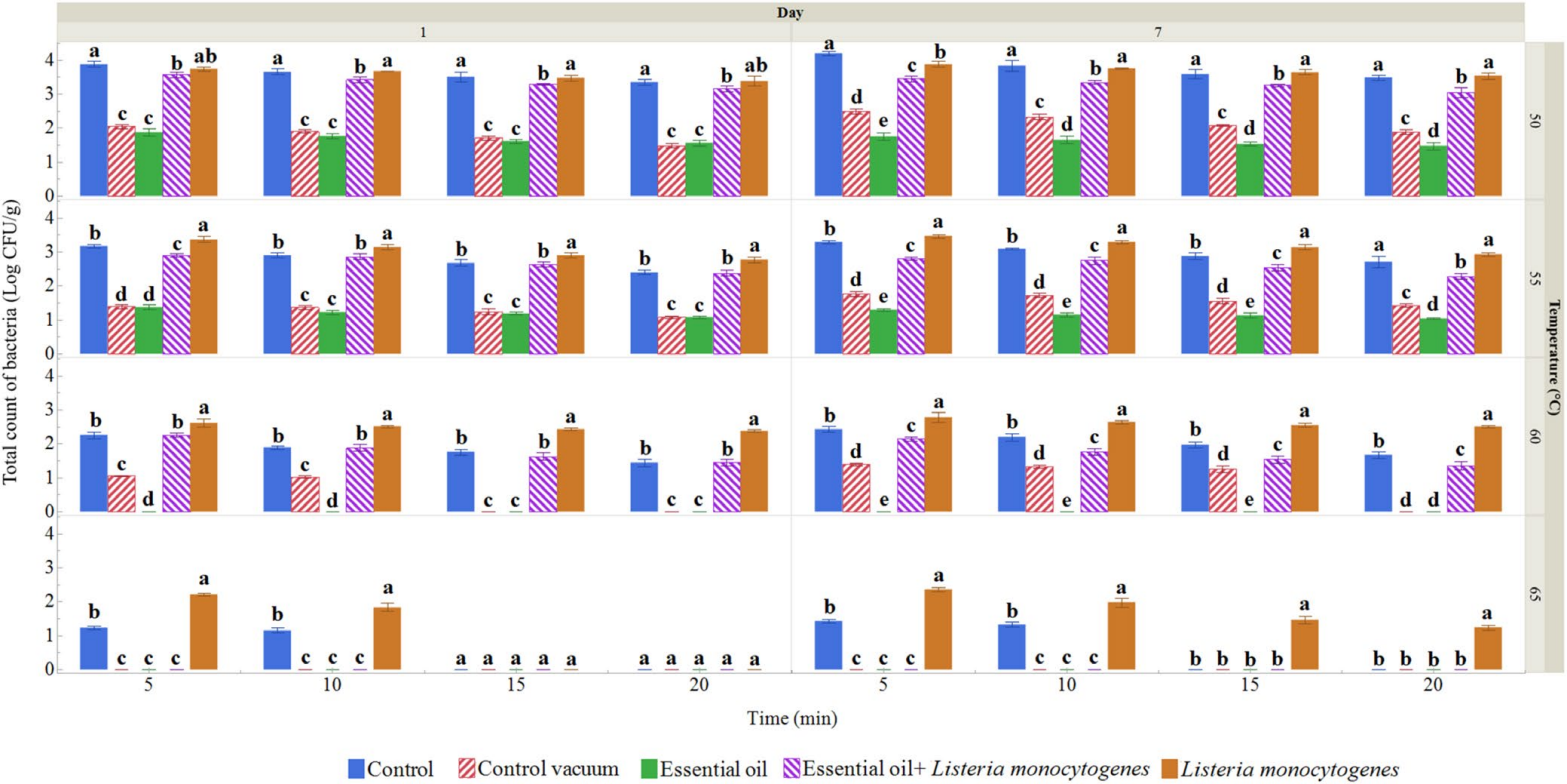
TVC (log CFU/g) in wild turkey sous vide samples treated at different temperatures and time, measured on the 1st and 7th day. Data represent the means of 3 samples, with bars indicating the standard deviation. Different letters within each group indicate significant differences (Tukey, *p* ≤ 0.05). Control: Wild turkey meat sample packed in polyethylene bags and stored at 4°C. Control vacuum: Wild turkey meat sample vacuum‐packed in polyethylene bags and stored at 4°C. Essential oil: Wild turkey meat sample treated with 10 mg/mL ROEO before being vacuum‐packed in polyethylene bags and stored at 4°C. *Listeria monocytogenes*: Wild turkey meat sample inoculated with *L. monocytogenes* before being vacuum‐packed in polyethylene bags and stored at 4°C. Essential oil + *Listeria monocytogenes* : Wild turkey meat sample treated with 10 mg/mL ROEO and *L. monocytogenes* before being vacuum‐packed in polyethylene bags and stored at 4°C.

Microbiological analysis of sous vide wild turkey samples continued on days 1 and 7. Figure 2 presents the total viable count (TVC) data. On day 1, the lowest temperature and shortest treatment times resulted in the highest TVC, ranging from 1.88 log CFU/g in the ROEO‐treated group to 3.88 log CFU/g in the control group. The ROEO‐treated group at 55°C for 20 min exhibited the lowest TVC, with a value of 1.08 log CFU/g, compared to the control group, which had a higher count at the same treatment condition. As the temperature increased, the bacterial count declined, with the highest numbers observed in the control group, particularly when exposed to temperatures up to 65°C for 10 min. At 65°C for 15 min, samples receiving ROEO plus heat had lower TVC than those exposed to heat alone, confirming an additional inhibitory effect of ROEO under these conditions. Similar patterns were seen in the ROEO‐treated and *L. monocytogenes* ‐inoculated groups, where bacterial counts were taken at 65°C for 10 min. These groups showed a significant decrease in TCB as the treatment time and temperature increased, particularly in the vacuum‐packed, temperature/time, and ROEO‐treated groups.

By day 7 of storage at 4°C, the temperature increased in all groups, except for the one treated with ROEO. In the control group, the total viable count (TVC) remained the same as on day 1, even at 65°C for 10 min. In the vacuum‐sealed control group, bacterial counts on day 7 were higher than on day 1 at the same temperature and duration. The group inoculated with *L. monocytogenes* showed elevated counts up to 65°C for 20 min, which indicated the worst TVC results for this group. For the ROEO‐treated group with *L. monocytogenes* inoculation, although TVC on day 7 was higher than on day 1, the count remained consistent up to the same temperature and time conditions. Based on these findings, it can be concluded that the ROEO‐treated group exhibited the most favourable trend in terms of TVC.

**FIGURE 3:**
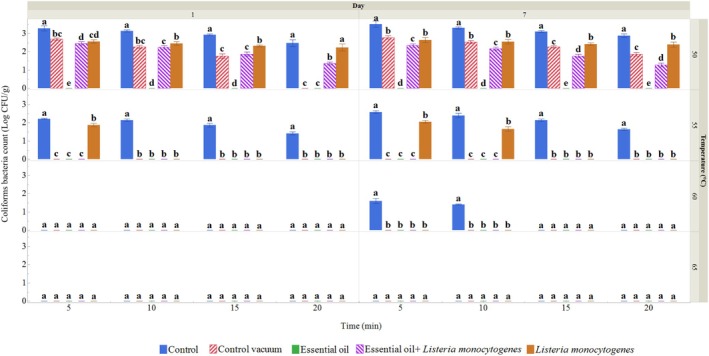
CB (log CFU/g) in wild turkey sous vide samples treated at different temperatures and time, measured on the 1st and 7th day. Data represents the means of 3 samples, with bars indicating the standard deviation. Different letters within each group indicate significant differences (Tukey, *p* ≤ 0.05). Control: Wild turkey meat sample packed in polyethylene bags and stored at 4°C. Control vacuum: Wild turkey meat sample vacuum‐packed in polyethylene bags and stored at 4°C. Essential oil: Wild turkey meat sample treated with 10 mg/mL ROEO before being vacuum‐packed in polyethylene bags and stored at 4°C. *Listeria monocytogenes*: Wild turkey meat sample inoculated with *L. monocytogenes* before being vacuum‐packed in polyethylene bags and stored at 4°C. Essential oil + *Listeria monocytogenes* : Wild turkey meat sample treated with 10 mg/mL ROEO and *L. monocytogenes* before being vacuum‐packed in polyethylene bags and stored at 4°C.

Following the experimental protocol, coliform bacteria (CB) were cultured on VRBL agar on both day 1 and day 7 (Figure 3). On day 1, no CBs were detected in the ROEO‐treated group, and their count was notably lower than the total microbial count. The highest CB count was observed in the untreated control group, with a value of 3.28 log CFU/g at the lowest temperature and time. The group with vacuum packaging had the lowest CB count, recorded at 2.70 log CFU/g, excluding the ROEO‐treated group. The control group showed the lowest CB count at 55°C for 20 min, while other groups had their lowest CB counts at lower temperatures and times. On day 7, CB counts at the lowest temperature and time ranged from 2.45 log CFU/g in the ROEO‐treated and *L. monocytogenes* (LM) inoculated groups to 3.52 log CFU/g in the control group. Similar to the total bacterial count (TBC), higher temperatures and longer treatment times led to higher CB counts in all groups except for the ROEO‐treated group. As on day 1, the control group showed the highest CB count at the lowest temperature and time, except for the ROEO‐treated group, where the count remained at zero. Similar to TBC, it was evident that vacuum treatment, EO, ROEO, and LM inoculation reduced CB contamination in the meat.

**FIGURE 4:**
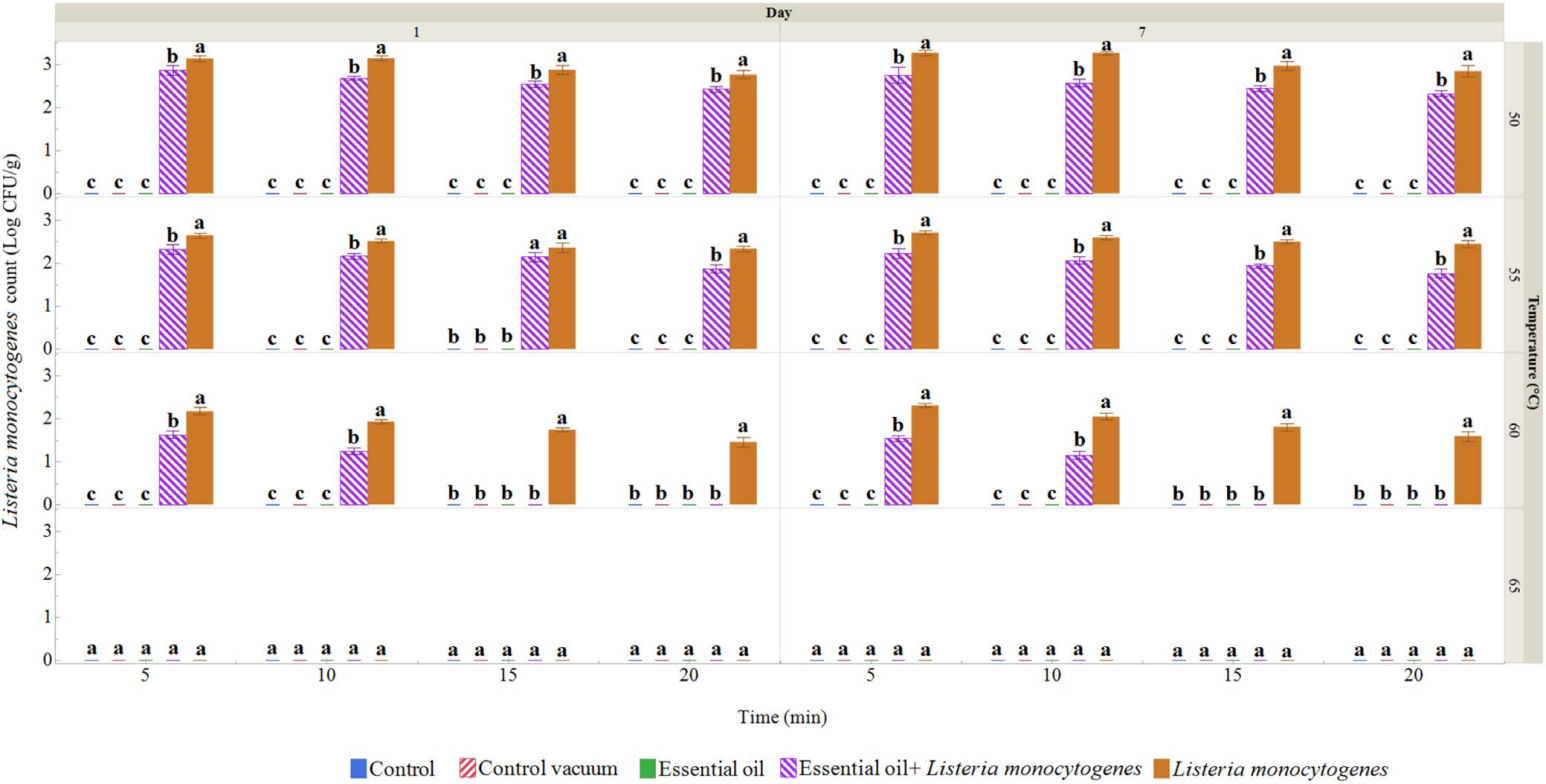
*Listeria monocytogenes* count (log CFU/g) in wild turkey sous vide samples treated at different temperatures and time, measured on the 1st and 7th day. Data represents the means of 3 samples, with bars indicating the standard deviation. Different letters within each group indicate significant differences (Tukey, *p* ≤ 0.05). Control: Wild turkey meat sample packed in polyethylene bags and stored at 4°C. Control vacuum: Wild turkey meat sample vacuum‐packed in polyethylene bags and stored at 4°C. Essential oil: Wild turkey meat sample treated with 10 mg/mL ROEO before being vacuum‐packed in polyethylene bags and stored at 4°C. *Listeria monocytogenes*: Wild turkey meat sample inoculated with *L. monocytogenes* before being vacuum‐packed in polyethylene bags and stored at 4°C. Essential oil + *Listeria monocytogenes* : Wild turkey meat sample treated with 10 mg/mL ROEO and *L. monocytogenes* before being vacuum‐packed in polyethylene bags and stored at 4°C.

*L. monocytogenes* (LM) was only found in the groups where it had been deliberately introduced. The highest microbial counts were observed in the sous vide wild turkey samples inoculated with LM at the lowest temperature and shortest exposure time (Figure 4). At 65°C for 15 min, the EO + *L. monocytogenes* group showed lower *L. monocytogenes* counts than the heat‐only inoculated group at the same condition. On day 1, the LM count was lowest in the inoculated groups at 60°C for 20 min (1.46 log CFU/g), while the lowest count for the ROEO‐treated groups occurred at 60°C for 10 min. Similar trends were observed on day 7, showing that the application of ROEO affected the growth rate of LM as the temperature and storage time increased. Even though LM was still present after 7 days, its concentration had decreased.

Vidaković Knežević et al. (2023) observed that there was no significant difference in LM levels on days 0 and 1 between control and EO‐treated samples of oregano and thyme. However, starting on day 2, EO‐treated oregano and thyme samples showed the first noticeable reduction in LM, with this decline continuing throughout the experiment. Our results also demonstrated a consistent decrease in LM levels over seven days with the use of EOs. Research by Hulankova and Borilova (2020) and Pateiro et al. (2021) has suggested that fats, proteins, and carbohydrates in food can bind essential oils (EOs), diminishing their bactericidal effectiveness. While the richer nutrient environment in foods, as compared to laboratory conditions, allows bacteria to recover more rapidly (Gill et al. 2002), the lower water content of food limits the antibacterial action of EOs.

In a study by Yuan et al. (2017), cinnamon EO inhibited LM growth when applied to the surface of smoked salmon, with the antimicrobial effect being dependent on both temperature and voltage. Furthermore, Pesavento et al. (2015) demonstrated that the effect of EOs on LM varies depending on the strain involved. In minced pork kept at 4°C, both oregano and thyme EOs significantly reduced the LM population. Our research supports these findings, indicating that low concentrations of EOs are effective in reducing LM levels.

The antimicrobial properties of cinnamon ( *Cinnamomum cassia* ) and clove ( *Syzygium aromaticum* ) EOs against foodborne pathogens like LM have also been reported by Khaleque et al. (2016). Their findings indicated that clove EO outperformed cinnamon EO in preventing bacterial growth in frozen and chilled minced meat. In contrast, only the highest concentration of cinnamon EO (5%) showed an effect, reducing *L. monocytogenes* growth by 12.5%–25%, depending on the storage conditions. Our study validated the effectiveness of a 1% EO concentration (10 mg/mL) in inhibiting LM growth.

### 3.5 Identification of Microbiota Isolated From Wild Turkey Sous Vide Meat

Meat that experiences rapid microbial proliferation may develop off‐flavours and/or discoloration, rendering it unsuitable for consumption. The competition between microorganisms in meat and the surrounding storage conditions plays a significant role in determining the diversity and makeup of the microbial communities present (Doulgeraki et al. 2012; Hyldgaard et al. 2015).

**FIGURE 5:**
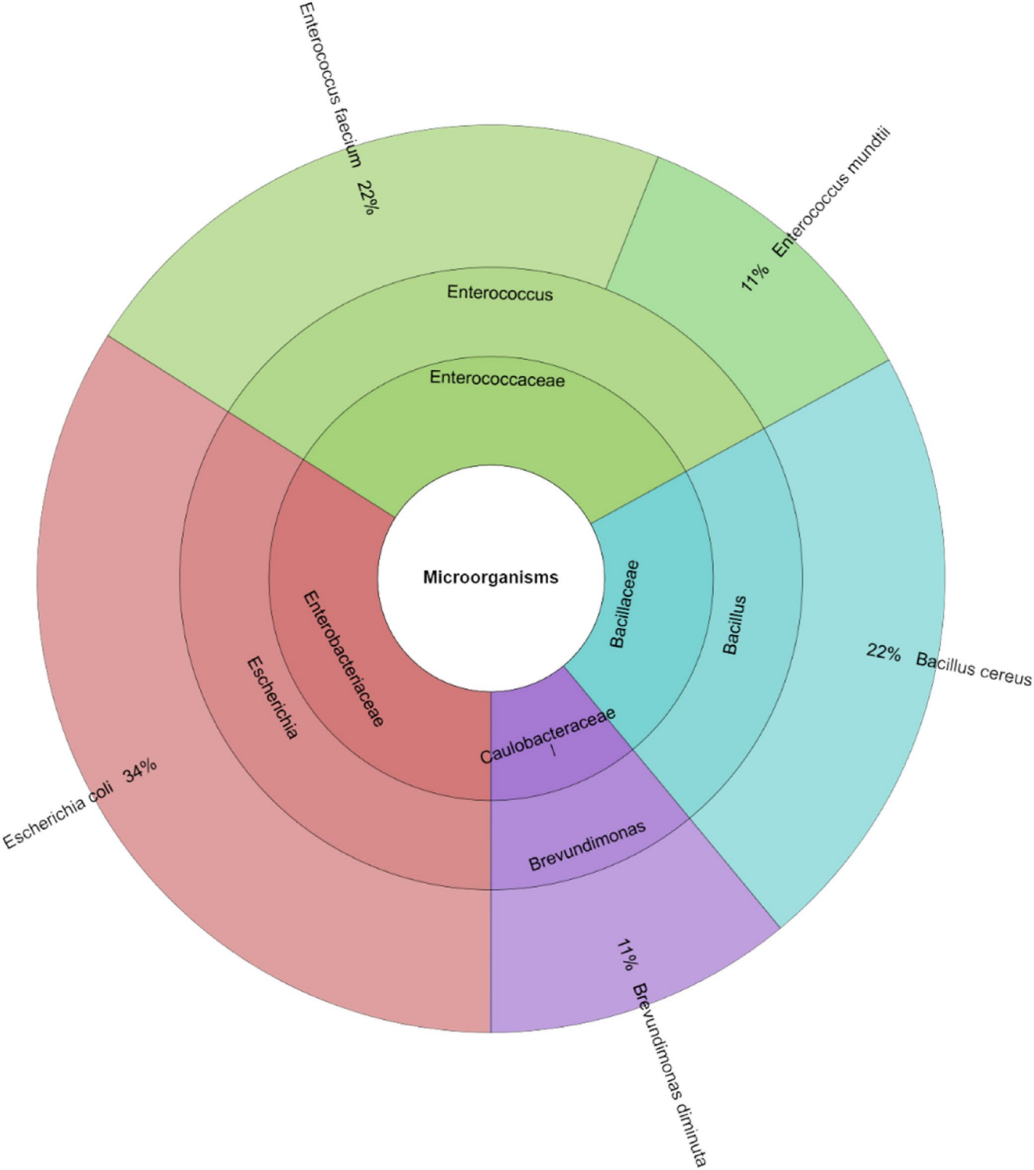
Individual taxa, genus and family isolated from wild turkey meat in day 0.

On day 0 of the observation period, only five bacterial species from four genera and four families were identified in the wild game meat samples. A total of 45 isolates were obtained from the pre‐treatment samples (Figure 5). The Enterobacteriaceae and Enterococcaceae families were most prevalent. *E. coli* was the most commonly isolated species, accounting for 34% of the isolates. In the subsequent section, we will compare the presence of each microorganism in the control and experimental groups after 1 and 7 days of storage.

**FIGURE 6:**
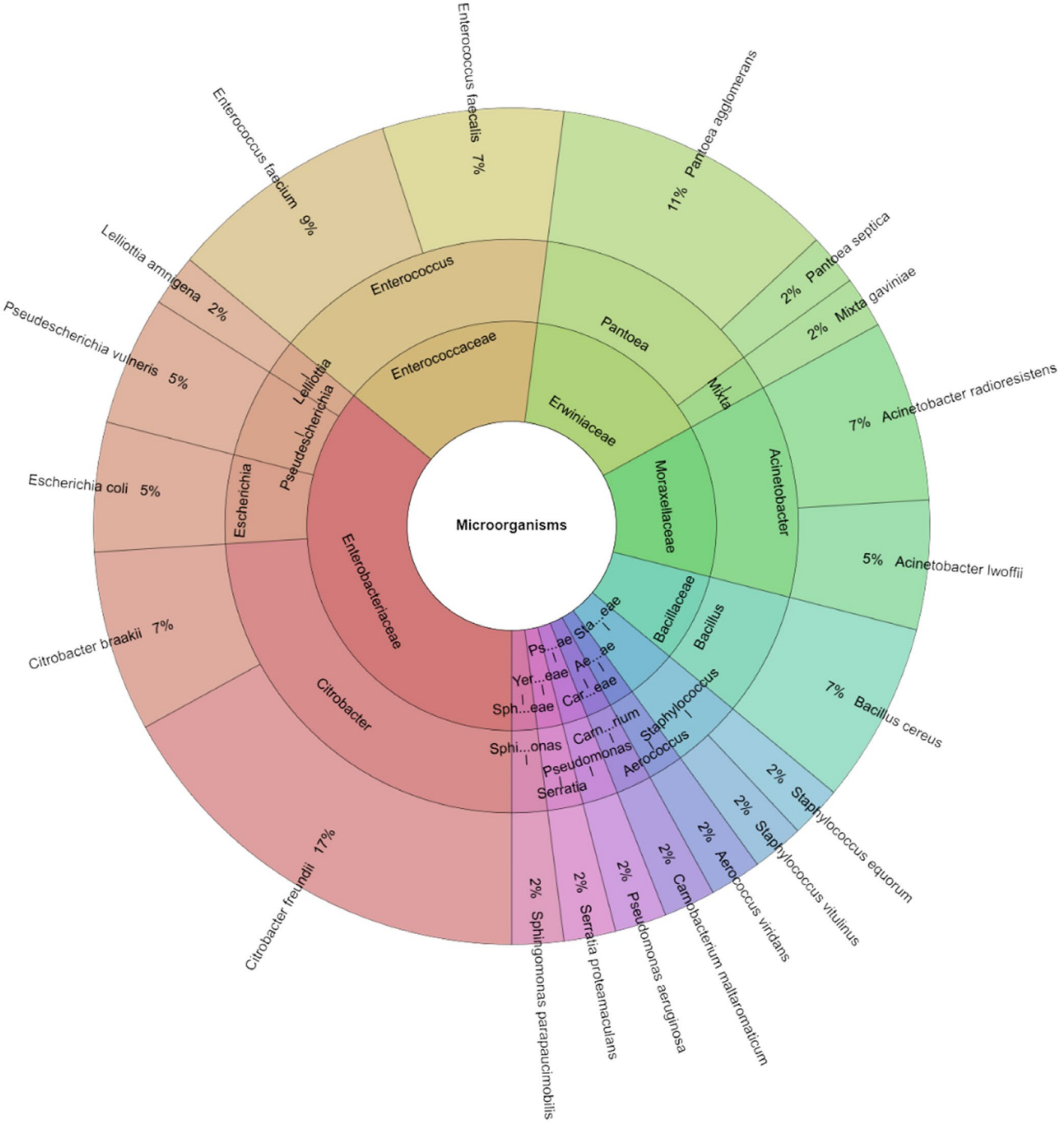
Individual taxa, genus and family isolated from wild turkey meat on day 1 in control groups.

On day 1 of evaluating the microbiological quality of sous vide wild turkey meat, a total of 20 bacterial species from 15 genera and 11 families (Figure 6) were identified in the control group. The most abundant species was *Citrobacter freundii* (17%), followed by *Pantoea agglomerans* (11%), *Enterococcus faecium* (9%), *Bacillus cereus* , and *Citrobacter braakii* (7%).

In a study by Palman et al. (2020) examining the microbiota of turkey meat, the majority of the microbial communities consisted of bacteria from the genera *Escherichia‐Shigella*, *Psychromonas*, *Pseudoalteromonas*, *Psychrobacter*, and *Pseudomonas*. At the start of the experiment, bacteria from the *Escherichia‐Shigella* group were abundant, but their numbers decreased by day 5 across all treatments. Bacteria from *Psychromonas*, *Pseudoalteromonas*, and *Psychrobacter* became more dominant, while *Pseudomonas* had a lower relative abundance. The control group, however, had a higher relative abundance of *Pseudomonas* and *Brochothrix*. Previous studies have shown that bacterial groups from Firmicutes, Proteobacteria, and Bacteroidetes are often linked to meat spoilage (Benson et al. 2014; Hyldgaard et al. 2015; Raimondi et al. 2018; Thomas et al. 2011). Palman et al. (2020) also confirmed the presence of *Pseudomonas* and *Brochothrix* in the control group, which aligns with our findings, where *Pseudomonas* was present. These microorganisms contribute to the spoilage of cooked meats during storage (Nychas et al. 2008) and in aerobically spoiled meat stored under refrigerated conditions (Kilcher et al. 2010; Russo et al. 2006). In particular, *Pseudomonas* has been consistently associated with spoilage in both this and other studies (Nychas et al. 2008). Other bacterial genera, such as *Citrobacter*, *Enterococcus*, and *Pantoea*, are potential spoilage organisms for poultry meat (Kilonzo‐Nthenge et al. 2013), and our results indicate that these bacterial communities were present in all of the samples throughout storage.

**FIGURE 7:**
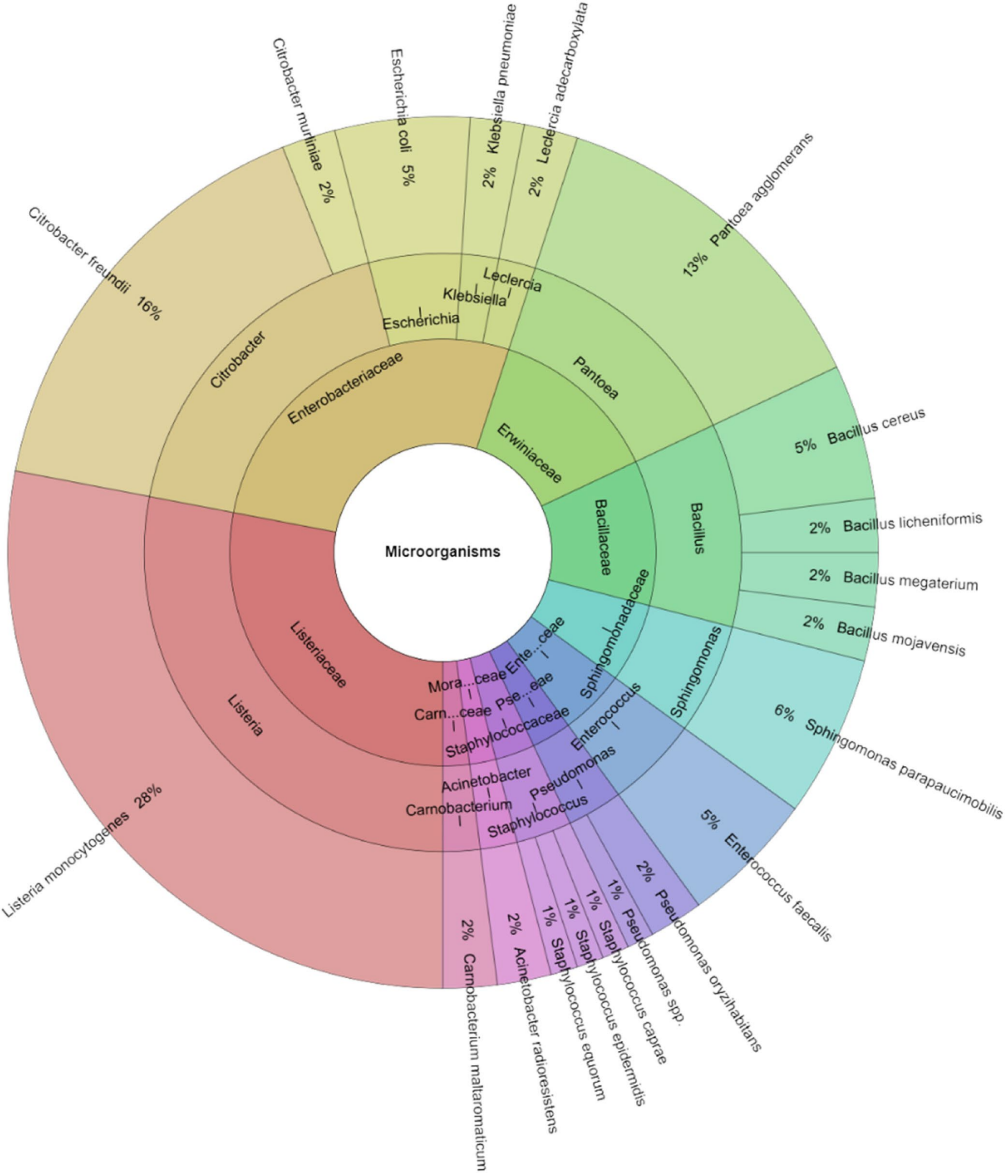
Individual taxa, genus, and family isolated from wild turkey meat in day 1 in experimental groups.

Following the control group, we focused on identifying the microbial composition in the experimental groups, where the same 20 species from 13 genera and 10 families were detected (Figure 7). In contrast to the control group, *L. monocytogenes* was the most frequently isolated bacterium from the sous vide wild turkey meat samples. After *L. monocytogenes* , the species most commonly isolated included *Citrobacter freundii* (16%), *Pantoea agglomerans* (13%), *Sphingomonas parapaucimobilis* (6%), *Bacillus cereus* , *Enterococcus faecalis* , and *Escherichia coli* (5%). Comparing these results with the control group, where *L. monocytogenes* was not present, we observe that the most commonly isolated species were similar to those in the experimental group. Specifically, *C. freundii* and *P. agglomerans* emerged as the most frequently detected species across all groups.

**FIGURE 8:**
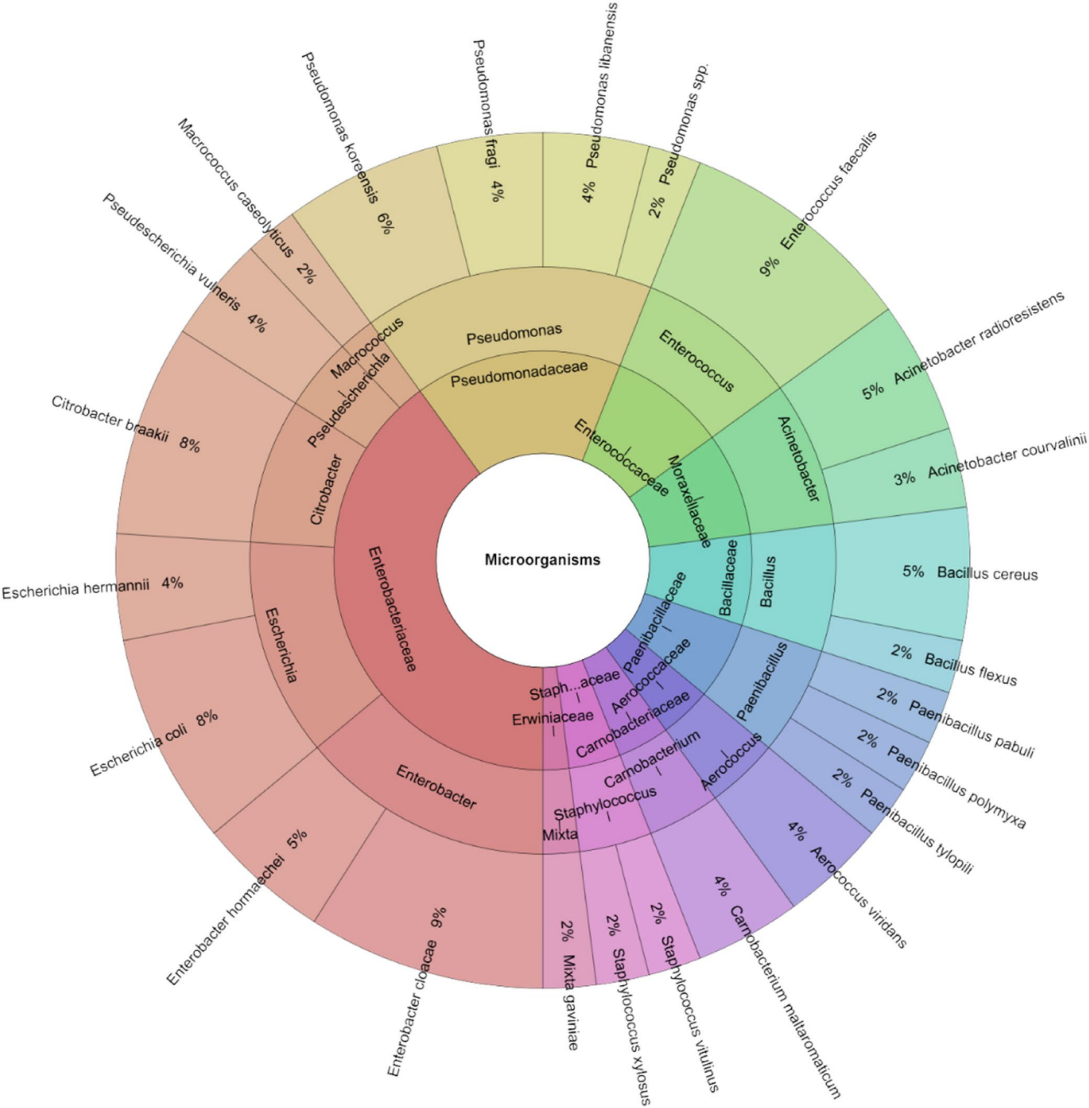
Individual taxa, genus and family isolated from wild turkey meat in day 7 in control groups.

At both the day 1 and day 7 marks, we assessed the diversity and number of microorganisms present in both the experimental and control groups (Figure 8). On day 7 of storage, the control group exhibited a higher variety of microbial species, with 24 species across 14 genera and 10 families, an increase from day 1. The most frequently detected species after 7 days were *Enterobacter cloacae* and *Enterococcus faecalis* (each at 9%), followed by *Citrobacter braakii* and *Escherichia coli* (both at 8%), and *Pantoea koreensis* at 6%.

**FIGURE 9:**
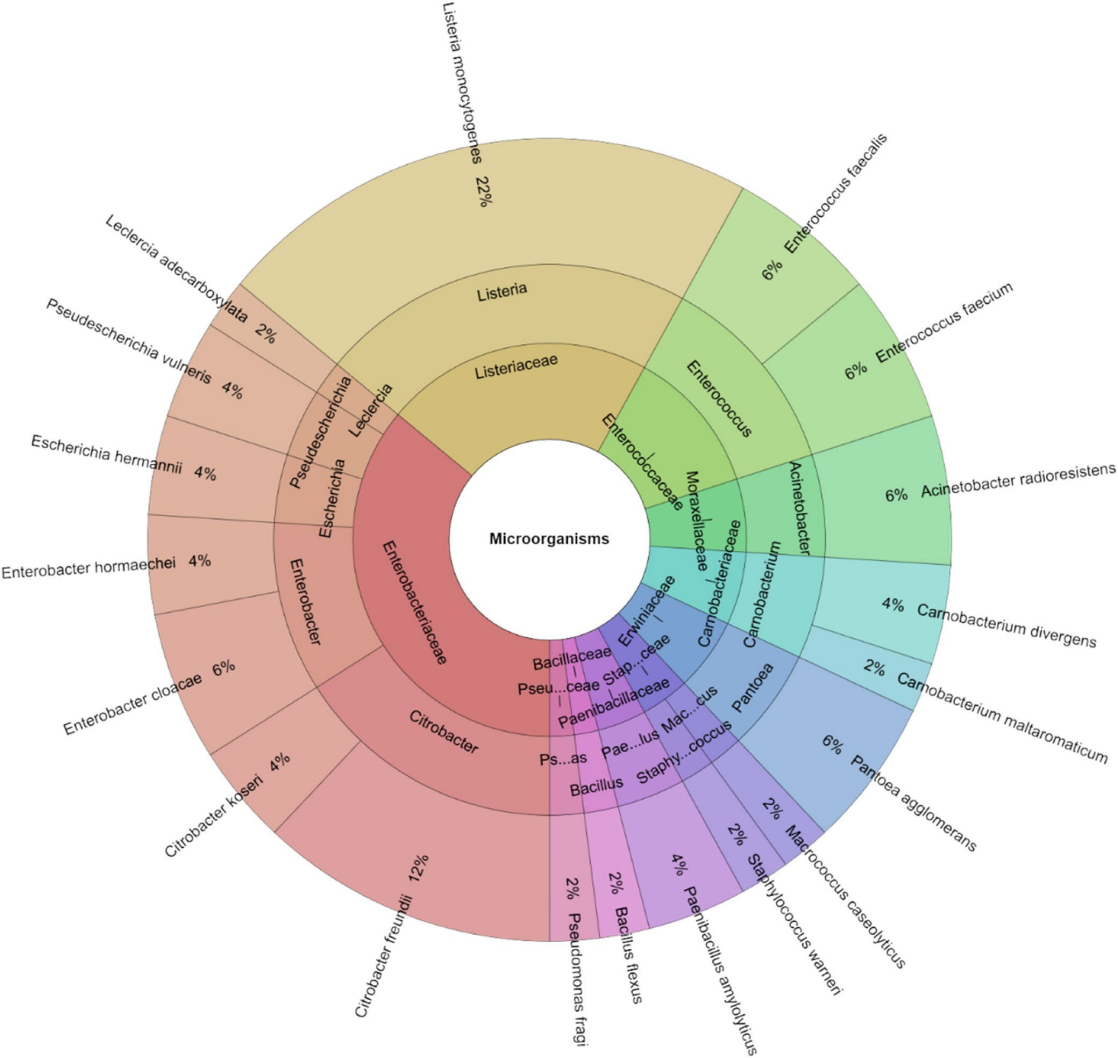
Krona chart: Species, genera and families isolated from wild turkey meat on day 7 in experimental groups.

After 7 days of storage, the experimental group exhibited fewer microbial species than on day 1 and also had a lower species count compared to the control group. As seen on day 1, *L. monocytogenes* remained the predominant bacterium in the experimental group. A total of 19 species were identified in the sous vide‐treated wild turkey samples, which is fewer than in the control group. These belonged to 15 genera and 11 families (Figure 9). The species most commonly found in the experimental group were *L. monocytogenes* (22%), *Citrobacter freundii* (12%), and other species, such as *Acinetobacter radioresistens* , *Enterobacter cloacae* , *Enterococcus faecalis* , *Enterococcus faecium* , and *Pantoea agglomerans* , all occurring at 6%.

The study by Augustyńska‐Prejsnar et al. (2021) on microbial identification in turkey meat using MALDI‐TOF MS Biotyper showed similar findings to ours, with a score value ≥ 2.00. In their analysis of 63 raw turkey meat samples, 7 families and 19 bacterial strains were identified, which aligns with our results. Additionally, 21 samples from roasted meat revealed 6 families and 10 bacterial strains. The marination process was found to reduce the diversity of bacterial families and mesophilic aerobic bacteria in both raw and roasted meat. In raw meat, four families were identified: Enterobacteriaceae, with *Enterobacter cloacae* being the most frequently isolated strain; Erwiniaceae, where *Pantoea agglomerans* dominated; Hafniaceae, with *Hafnia alvei* ; and Pseudomonadaceae, with *Pseudomonas putida* as the predominant strain (Augustyńska‐Prejsnar et al. 2021).

## 4 Conclusion

The literature indicates that research on wild turkey meat has been limited. Based on both existing literature and our study, this research is unique in examining the antimicrobial effects of rosemary essential oil (ROEO) on *L. monocytogenes* under in situ conditions in sous‐vide wild turkey meat. Additionally, it explores the microbiological quality of meat treated with ROEO and samples inoculated with *L. monocytogenes* . The chemical composition of ROEO included 1,8‐cineole, α‐pinene, camphor, and β‐pinene, which are associated with antimicrobial activity and likely act additively/synergistically; this is consistent with our observed inhibition of *L. monocytogenes* . Our results consistently demonstrate the antimicrobial effect of ROEO on *L. monocytogenes* in sous vide turkey meat. Furthermore, differences in bacterial abundance, as identified by mass spectrometry, were evident. Overall, the findings confirm that ROEO possesses antimicrobial activity against *L. monocytogenes* in this model, making it a promising preservative for use in sous‐vide turkey meat, especially during storage over a 7‐day period. These findings apply exclusively to *Listeria monocytogenes* in the sous‐vide wild turkey model and do not constitute direct evidence of ROEO effects on other pathogens.

## Author Contributions

**Miroslava Kačániová:** methodology, investigation, writing – original draft, formal analyses, supervision, project administration, funding acquisition, visualisation. **Guiguo Zhang:** formal analyses, writing – review and editing. **Suzana Popovic:** formal analyses, methodology, writing – original draft. **Alessandro Bianchi:** formal analyses, methodology, writing – original draft. **Zhaojun Ban:** formal analyses, writing – review and editing. **Li Li:** formal analyses, writing – review and editing. **Natália Čmiková:** formal analyses, writing – review and editing. **Joel Horacio Elizondo‐Luevano:** formal analyses, writing – review and editing. **Anis Ben Hsouna:** formal analyses, writing – review and editing. **Rania Ben Saad:** formal analyses, writing – review and editing. **Peter Haščík:** formal analyses, writing – review and editing. **Stefania Garzoli:** formal analyses, writing – review and editing, supervision.

## Funding

This work was supported by The potential of the essential oils from aromatic plants for medical use and food preservation, APVV‐20‐0058; Chemical properties and biological activity (in vitro, in vivo and in situ) of plant volatile mixtures, their main components and inclusion systems, VEGA 1/0059/24; Ministry of Science, Technological Development, and Innovations of the Republic of Serbia, No. 451‐03‐66/2024‐03/200122, No. 451‐03‐65/2024‐03/200122.

## Conflicts of Interest

The authors declare no conflicts of interest.

## Data Availability

The data that support the findings of this study are available from the corresponding author upon reasonable request.

## References

[emi470299-bib-0001] Adams, R. P. 2007. Identification of Essential Oil Components by Gas Chromatography/Mass Spectroscopy. Allured Publishing Corporation.

[emi470299-bib-0002] Angioni, A. , A. Barra , E. Cereti , et al. 2004. “Chemical Composition, Plant Genetic Differences, Antimicrobial and Antifungal Activity Investigation of the Essential Oil of *Rosmarinus officinalis* L.” Journal of Agricultural and Food Chemistry 52: 3530–3535. 10.1021/jf049913t. 15161226

[emi470299-bib-0003] Augustyńska‐Prejsnar, A. , P. Hanus , Z. Sokołowicz , and M. Kačániová . 2021. “Assessment of Technological Characteristics and Microbiological Quality of Marinated Turkey Meat With the Use of Dairy Products and Lemon Juice.” Animal Bioscience 34: 2003–2011. 10.5713/ab.21.0120. 34293846 PMC8563228

[emi470299-bib-0004] Autio, T. , T. Säteri , M. Fredriksson‐Ahomaa , M. Rahkio , J. Lundén , and H. Korkeala . 2000. “ *Listeria monocytogenes Contamination Pattern in Pig Slaughterhouses* .” Journal of Food Protection 63: 1438–1442. 10.4315/0362-028X-63.10.1438. 11041148

[emi470299-bib-0005] Baldwin, D. 2021. “Sous Vide Cooking.” In Handbook of Molecular Gastronomy, edited by R. M. Burke , A. L. Kelly , C. Lavelle , and H. T. V. Kientza , First ed., 531–535. CRC Press. 10.1201/9780429168703-80.

[emi470299-bib-0006] Ballester‐Costa, C. , E. Sendra , J. Fernández‐López , J. A. Pérez‐Álvarez , and M. Viuda‐Martos . 2013. “Chemical Composition and In Vitro Antibacterial Properties of Essential Oils of Four Thymus Species From Organic Growth.” Industrial Crops and Products 50: 304–311. 10.1016/j.indcrop.2013.07.052.

[emi470299-bib-0007] Barbuti, S. , and G. Parolari . 2002. “Validation of Manufacturing Process to Control Pathogenic Bacteria in Typical Dry Fermented Products.” Meat Science 62: 323–329. 10.1016/S0309-1740(02)00124-9. 22061608

[emi470299-bib-0008] Becer, E. , E. M. Altundağ , M. Güran , et al. 2023. “Composition and Antibacterial, Anti‐Inflammatory, Antioxidant, and Anticancer Activities of *Rosmarinus officinalis* L. Essential Oil.” South African Journal of Botany 160: 437–445. 10.1016/j.sajb.2023.07.028.

[emi470299-bib-0009] Benson, A. K. , J. R. D. David , S. E. Gilbreth , et al. 2014. “Microbial Successions Are Associated With Changes in Chemical Profiles of a Model Refrigerated Fresh Pork Sausage During an 80‐Day Shelf Life Study.” Applied and Environmental Microbiology 80: 5178–5194. 10.1128/AEM.00774-14. 24928886 PMC4136103

[emi470299-bib-0010] Bortolaia, V. , C. Espinosa‐Gongora , and L. Guardabassi . 2016. “Human Health Risks Associated With Antimicrobial‐Resistant Enterococci and *Staphylococcus aureus* on Poultry Meat.” Clinical Microbiology and Infection 22: 130–140. 10.1016/j.cmi.2015.12.003. 26706616

[emi470299-bib-0011] Bozin, B. , N. Mimica‐Dukic , I. Samojlik , and E. Jovin . 2007. “Antimicrobial and Antioxidant Properties of Rosemary and Sage (*Rosmarinus officinalis* L. and *Salvia officinalis* L., Lamiaceae) Essential Oils.” Journal of Agricultural and Food Chemistry 55: 7879–7885. 10.1021/jf0715323. 17708648

[emi470299-bib-0012] Burt, S. 2004. “Essential Oils: Their Antibacterial Properties and Potential Applications in Foods—A Review.” International Journal of Food Microbiology 94: 223–253. 10.1016/j.ijfoodmicro.2004.03.022. 15246235

[emi470299-bib-0013] Cacciatore, F. A. , C. Maders , B. Alexandre , C. M. Barreto Pinilla , A. Brandelli , and P. Da Silva Malheiros . 2022. “Carvacrol Encapsulation Into Nanoparticles Produced From Chia and Flaxseed Mucilage: Characterization, Stability and Antimicrobial Activity Against Salmonella and *Listeria monocytogenes* .” Food Microbiology 108: 104116. 10.1016/j.fm.2022.104116. 36088121

[emi470299-bib-0014] Calín‐Sánchez, Á. , A. Szumny , A. Figiel , K. Jałoszyński , M. Adamski , and Á. A. Carbonell‐Barrachina . 2011. “Effects of Vacuum Level and Microwave Power on Rosemary Volatile Composition During Vacuum–Microwave Drying.” Journal of Food Engineering 103: 219–227. 10.1016/j.jfoodeng.2010.10.018.

[emi470299-bib-0015] Chauhan, K. , and A. Rao . 2024. “Clean‐Label Alternatives for Food Preservation: An Emerging Trend.” Heliyon 10: e35815. 10.1016/j.heliyon.2024.e35815. 39247286 PMC11379619

[emi470299-bib-0016] Coimbra, A. , F. Carvalho , A. P. Duarte , and S. Ferreira . 2022. “Antimicrobial Activity of Thymus Zygis Essential Oil Against Listeria Monocytogenes and Its Application as Food Preservative.” Innovative Food Science & Emerging Technologies 80: 103077. 10.1016/j.ifset.2022.103077.

[emi470299-bib-0017] Colak, H. , H. Hampikyan , B. Ulusoy , and E. B. Bingol . 2007. “Presence of *Listeria monocytogenes* in Turkish Style Fermented Sausage (Sucuk).” Food Control 18: 30–32. 10.1016/j.foodcont.2005.08.003.

[emi470299-bib-0018] Dias Costa, R. , V. Silva , A. Leite , et al. 2023. “Salmonella spp., *Escherichia Coli* and Enterobacteriaceae Control at a Pig Abattoir: Are we Missing Lairage Time Effect, Pig Skin, and Internal Carcass Surface Contamination?” Food 12: 2910. 10.3390/foods12152910. PMC10418833 37569179

[emi470299-bib-0019] Diass, K. , M. Merzouki , K. Elfazazi , et al. 2023. “Essential Oil of *Lavandula officinalis*: Chemical Composition and Antibacterial Activities.” Plants 12: 1571. 37050197 10.3390/plants12071571 PMC10097330

[emi470299-bib-0020] Díaz‐Maroto, M. C. , M. S. Pérez‐Coello , E. Sánchez‐Palomo , and M. A. González Viñas . 2007. “Impact of Drying and Storage Time on Sensory Characteristics of Rosemary (*Rosmarinus officinalis* l.).” Journal of Sensory Studies 22: 34–48. 10.1111/j.1745-459X.2007.00093.x.

[emi470299-bib-0021] Doulgeraki, A. I. , D. Ercolini , F. Villani , and G.‐J. E. Nychas . 2012. “Spoilage Microbiota Associated to the Storage of Raw Meat in Different Conditions.” International Journal of Food Microbiology 157: 130–141. 10.1016/j.ijfoodmicro.2012.05.020. 22682877

[emi470299-bib-0022] Duda‐Chodak, A. , T. Tarko , and K. Petka‐Poniatowska . 2023. “Antimicrobial Compounds in Food Packaging.” International Journal of Molecular Sciences 24: 2457. 10.3390/ijms24032457. 36768788 PMC9917197

[emi470299-bib-0023] Figueiredo, A. C. , J. G. Barroso , L. G. Pedro , and J. J. C. Scheffer . 2008. “Factors Affecting Secondary Metabolite Production in Plants: Volatile Components and Essential Oils.” Flavour and Fragrance Journal 23: 213–226. 10.1002/ffj.1875.

[emi470299-bib-0024] Flamini, G. , P. L. Cioni , I. Morelli , M. Macchia , and L. Ceccarini . 2002. “Main Agronomic−Productive Characteristics of Two Ecotypes of *Rosmarinus officinalis* L. and Chemical Composition of Their Essential Oils.” Journal of Agricultural and Food Chemistry 50: 3512–3517. 10.1021/jf011138j. 12033820

[emi470299-bib-0025] Ghabraie, M. , K. D. Vu , L. Tata , S. Salmieri , and M. Lacroix . 2016. “Antimicrobial Effect of Essential Oils in Combinations Against Five Bacteria and Their Effect on Sensorial Quality of Ground Meat.” LWT ‐ Food Science and Technology 66: 332–339. 10.1016/j.lwt.2015.10.055.

[emi470299-bib-0026] Gill, A. O. , P. Delaquis , P. Russo , and R. A. Holley . 2002. “Evaluation of Antilisterial Action of Cilantro Oil on Vacuum Packed Ham.” International Journal of Food Microbiology 73: 83–92. 10.1016/S0168-1605(01)00712-7. 11883677

[emi470299-bib-0027] Guo, J. , X. Hu , Z. Gao , et al. 2021. “Global Transcriptomic Response of *Listeria monocytogenes* Exposed to Fingered Citron (*Citrus medica* L. Var. *Sarcodactylis swingle*) Essential Oil.” Food Research International 143: 110274. 10.1016/j.foodres.2021.110274. 33992374

[emi470299-bib-0028] Höll, L. , J. Behr , and R. F. Vogel . 2016. “Identification and Growth Dynamics of Meat Spoilage Microorganisms in Modified Atmosphere Packaged Poultry Meat by MALDI‐TOF MS.” Food Microbiology 60: 84–91. 10.1016/j.fm.2016.07.003. 27554149

[emi470299-bib-0029] Hulankova, R. , and G. Borilova . 2020. “Modeling Dependence of Growth Inhibition of Salmonella Typhimurium and *Listeria monocytogenes* by Oregano or Thyme Essential Oils on the Chemical Composition of Minced Pork.” Journal of Food Safety 40: e12818. 10.1111/jfs.12818.

[emi470299-bib-0030] Hyldgaard, M. , R. L. Meyer , M. Peng , et al. 2015. “Binary Combination of Epsilon‐Poly‐l‐Lysine and Isoeugenol Affect Progression of Spoilage Microbiota in Fresh Turkey Meat, and Delay Onset of Spoilage in *Pseudomonas putida* Challenged Meat.” International Journal of Food Microbiology 215: 131–142. 10.1016/j.ijfoodmicro.2015.09.014. 26433458

[emi470299-bib-0031] Jaber, H. , R. Ijoub , A. Zaher , et al. 2017. “Microbiological Study of Turkey Meat Marketed in Kenitra (North‐Oust of Morocco).” Journal of Nutrition & Food Sciences 7: 1000620. 10.4172/2155-9600.1000620.

[emi470299-bib-0032] Kačániová, M. , N. Čmiková , M. I. Kluz , et al. 2024. “Anti‐Salmonella Activity of *Thymus serpyllum* Essential Oil in Sous Vide Cook–Chill Rabbit Meat.” Food 13: 200. 10.3390/foods13020200. PMC10815041 38254501

[emi470299-bib-0033] Kačániová, M. , S. Garzoli , A. Ben Hsouna , et al. 2024. “Enhancing Deer Sous Vide Meat Shelf Life and Safety With *Eugenia caryophyllus* Essential Oil Against *Salmonella enterica* .” Food 13: 2512. 10.3390/foods13162512. PMC11353597 39200440

[emi470299-bib-0034] Keba, A. , M. L. Rolon , A. Tamene , et al. 2020. “Review of the Prevalence of Foodborne Pathogens in Milk and Dairy Products in Ethiopia.” International Dairy Journal 109: 104762. 10.1016/j.idairyj.2020.104762. 33013007 PMC7430047

[emi470299-bib-0035] Khaleque, M. A. , C. A. Keya , K. N. Hasan , M. M. Hoque , Y. Inatsu , and M. L. Bari . 2016. “Use of Cloves and Cinnamon Essential Oil to Inactivate *Listeria monocytogenes* in Ground Beef at Freezing and Refrigeration Temperatures.” LWT 74: 219–223. 10.1016/j.lwt.2016.07.042.

[emi470299-bib-0036] Kilcher, S. , M. J. Loessner , and J. Klumpp . 2010. “ *Brochothrix thermosphacta* Bacteriophages Feature Heterogeneous and Highly Mosaic Genomes and Utilize Unique Prophage Insertion Sites.” Journal of Bacteriology 192: 5441–5453. 10.1128/JB.00709-10. 20709901 PMC2950505

[emi470299-bib-0037] Kilonzo‐Nthenge, A. , E. Rotich , and S. N. Nahashon . 2013. “Evaluation of Drug‐Resistant Enterobacteriaceae in Retail Poultry and Beef.” Poultry Science 92: 1098–1107. 10.3382/ps.2012-02581. 23472034

[emi470299-bib-0038] Kunert‐Filho, H. C. , T. Q. Furian , R. Sesterhenn , et al. 2022. “Bacterial Community Identification in Poultry Carcasses Using High‐Throughput Next Generation Sequencing.” International Journal of Food Microbiology 364: 109533. 10.1016/j.ijfoodmicro.2022.109533. 35066330

[emi470299-bib-0039] Lin, C.‐M. , K. Takeuchi , L. Zhang , et al. 2006. “Cross‐Contamination Between Processing Equipment and Deli Meats by *Listeria monocytogenes* .” Journal of Food Protection 69: 71–79. 10.4315/0362-028X-69.1.71. 16416903

[emi470299-bib-0040] Machado, C. C. , W. P. Silvestre , L. B. A. Touguinha , G. F. Pauletti , and J. Schwambach . 2024. “Use of *Rosmarinus officinalis* Essential Oil and Its Fractions in the Alternative Control of Grapevine Phytopathogens.” Brazilian Archives of Biology and Technology 67: e24240022. 10.1590/1678-4324-2024240022.

[emi470299-bib-0041] Martins, E. A. , and P. M. Leal Germano . 2011. “ *Listeria monocytogenes* in Ready‐To‐Eat, Sliced, Cooked Ham and Salami Products, Marketed in the City of São Paulo, Brazil: Occurrence, Quantification, and Serotyping.” Food Control 22: 297–302. 10.1016/j.foodcont.2010.07.026.

[emi470299-bib-0042] Mazzarrino, G. , A. Paparella , C. Chaves‐López , et al. 2015. “ *Salmonella enterica* and *Listeria monocytogenes* Inactivation Dynamics After Treatment With Selected Essential Oils.” Food Control 50: 794–803. 10.1016/j.foodcont.2014.10.029.

[emi470299-bib-0043] Mouahid, A. , C. Dufour , and E. Badens . 2017. “Supercritical CO_2_ Extraction From Endemic Corsican Plants; Comparison of Oil Composition and Extraction Yield With Hydrodistillation Method.” Journal of CO2 Utilization 20: 263–273. 10.1016/j.jcou.2017.06.003.

[emi470299-bib-0044] Naveena, B. M. , M. Muthukumar , A. R. Sen , Y. Babji , and T. R. K. Murthy . 2006. “Improvement of Shelf‐Life of Buffalo Meat Using Lactic Acid, Clove Oil and Vitamin C During Retail Display.” Meat Science 74: 409–415. 10.1016/j.meatsci.2006.04.020. 22062853

[emi470299-bib-0045] Nychas, G.‐J. E. , P. N. Skandamis , C. C. Tassou , and K. P. Koutsoumanis . 2008. “Meat Spoilage During Distribution.” Meat Science 78: 77–89. 10.1016/j.meatsci.2007.06.020. 22062098

[emi470299-bib-0046] Oakley, B. B. , C. A. Morales , J. Line , et al. 2013. “The Poultry‐Associated Microbiome: Network Analysis and Farm‐To‐Fork Characterizations.” PLoS One 8: e57190. 10.1371/journal.pone.0057190. 23468931 PMC3584146

[emi470299-bib-0047] Oliveira, M. , N. M. Pedroso , T. Sales‐Luís , M. Santos‐Reis , L. Tavares , and C. L. Vilela . 2010. “Antimicrobial‐Resistant Salmonella Isolated From Eurasian Otters (*Lutra lutra* Linnaeus, 1758) in Portugal.” Journal of Wildlife Diseases 46: 1257–1261. 10.7589/0090-3558-46.4.1257. 20966276

[emi470299-bib-0048] Oualdi, I. , K. Diass , S. Azizi , et al. 2023. “ *Rosmarinus officinalis* Essential Oils From Morocco: New Advances on Extraction, GC/MS Analysis, and Antioxidant Activity.” Natural Product Research 37: 2003–2008. 10.1080/14786419.2022.2111561. 35959692

[emi470299-bib-0049] Özcan, M. M. , and J.‐C. Chalchat . 2008. “Chemical Composition and Antifungal Activity of Rosemary (*Rosmarinus officinalis* L.) Oil From Turkey.” International Journal of Food Sciences and Nutrition 59: 691–698. 10.1080/09637480701777944. 18654909

[emi470299-bib-0050] Palman, Y. , R. De Leo , A. Pulvirenti , S. J. Green , and Z. Hayouka . 2020. “Antimicrobial Peptide Cocktail Activity in Minced Turkey Meat.” Food Microbiology 92: 103580. 10.1016/j.fm.2020.103580. 32950164

[emi470299-bib-0051] Pascual, C. , T. P. Robinson , M. J. Ocio , O. O. Aboaba , and B. M. Mackey . 2001. “The Effect of Inoculum Size and Sublethal Injury on the Ability of *Listeria monocytogenes* to Initiate Growth Under Suboptimal Conditions.” Letters in Applied Microbiology 33: 357–361. 10.1046/j.1472-765X.2001.01012.x. 11696096

[emi470299-bib-0052] Pateiro, M. , P. E. S. Munekata , A. S. Sant'Ana , R. Domínguez , D. Rodríguez‐Lázaro , and J. M. Lorenzo . 2021. “Application of Essential Oils as Antimicrobial Agents Against Spoilage and Pathogenic Microorganisms in Meat Products.” International Journal of Food Microbiology 337: 108966. 10.1016/j.ijfoodmicro.2020.108966. 33202297

[emi470299-bib-0053] Pauli, A. , and H. Schilcher . 2010. “12 In Vitro Antimicrobial Activities of Essential Oils Monographed in the European Pharmacopoeia 6th Edition.” ESSENTIAL 353.

[emi470299-bib-0054] Paulsen, P. 2011. “Hygiene and Microbiology of Meat From Wild Game: An Austrian View.” In Game Meat Hygiene in Focus, edited by P. Paulsen , A. Bauer , M. Vodnansky , R. Winkelmayer , and F. J. M. Smulders , 19–37. Wageningen Academic Publishers. 10.3920/978-90-8686-723-3_1.

[emi470299-bib-0055] Pesavento, G. , C. Calonico , A. R. Bilia , et al. 2015. “Antibacterial Activity of Oregano, Rosmarinus and Thymus Essential Oils Against Staphylococcus Aureus and *Listeria monocytogenes* in Beef Meatballs.” Food Control 54: 188–199. 10.1016/j.foodcont.2015.01.045.

[emi470299-bib-0056] Rafya, M. , N. Zehhar , A. Hafidi , and F. Benkhalti . 2024. “Review of *Rosmarinus officinalis* L. Essential Oil, Hydrosol, and Residues Analysis: Composition, Bioactivities, and Valorization.” Industrial Crops and Products 221: 119392. 10.1016/j.indcrop.2024.119392.

[emi470299-bib-0057] Raimondi, S. , M. R. Nappi , T. M. Sirangelo , et al. 2018. “Bacterial Community of Industrial Raw Sausage Packaged in Modified Atmosphere Throughout the Shelf Life.” International Journal of Food Microbiology 280: 78–86. 10.1016/j.ijfoodmicro.2018.04.041. 29783046

[emi470299-bib-0058] Rašković, A. , I. Milanović , N. Pavlović , T. Ćebović , S. Vukmirović , and M. Mikov . 2014. “Antioxidant Activity of Rosemary (*Rosmarinus officinalis* L.) Essential Oil and Its Hepatoprotective Potential.” BMC Complementary and Alternative Medicine 14: 225. 10.1186/1472-6882-14-225. 25002023 PMC4227022

[emi470299-bib-0059] Rasooli, I. 2008. “Antimycotoxigenic Characteristics of *Rosmarinus officinalis* and *Trachyspermum copticum* L. Essential Oils.” International Journal of Food Microbiology 122: 135–139. 10.1016/j.ijfoodmicro.2007.11.048. 18190993

[emi470299-bib-0060] Rasooli, I. , M. B. Rezaei , and A. Allameh . 2006. “Ultrastructural Studies on Antimicrobial Efficacy of Thyme Essential Oils on *Listeria monocytogenes* .” International Journal of Infectious Diseases 10: 236–241. 10.1016/j.ijid.2005.05.006. 16412677

[emi470299-bib-0061] Rathore, S. , S. Mukhia , S. Kapoor , V. Bhatt , R. Kumar , and R. Kumar . 2022. “Seasonal Variability in Essential Oil Composition and Biological Activity of *Rosmarinus officinalis* L. Accessions in the Western Himalaya.” Scientific Reports 12: 3305. 10.1038/s41598-022-07298-x. 35228638 PMC8885650

[emi470299-bib-0062] Rout, S. , S. Tambe , R. K. Deshmukh , et al. 2022. “Recent Trends in the Application of Essential Oils: The Next Generation of Food Preservation and Food Packaging.” Trends in Food Science & Technology 129: 421–439. 10.1016/j.tifs.2022.10.012.

[emi470299-bib-0063] Russo, F. , D. Ercolini , G. Mauriello , and F. Villani . 2006. “Behaviour of *Brochothrix thermosphacta* in Presence of Other Meat Spoilage Microbial Groups.” Food Microbiology 23: 797–802. 10.1016/j.fm.2006.02.004. 16943085

[emi470299-bib-0064] Sabbahi, M. , A. Tahani , A. Talhaoui , and A. El‐Bachiri . 2023. “Cartography and Chemical Profiling of Rosemary Essential Oil in Eastern High Atlas Mountains, Morocco.” Materials Today: Proceedings 72: 3435–3441. 10.1016/j.matpr.2022.08.087.

[emi470299-bib-0065] Saenz‐García, C. E. , P. Castañeda‐Serrano , E. M. Mercado Silva , C. Z. Alvarado , and G. M. Nava . 2020. “Insights Into the Identification of the Specific Spoilage Organisms in Chicken Meat.” Food 9: 225. 10.3390/foods9020225. PMC7073905 32093245

[emi470299-bib-0066] Sakar, E. H. , A. Zeroual , A. Kasrati , and S. Gharby . 2023. “Combined Effects of Domestication and Extraction Technique on Essential Oil Yield, Chemical Profiling, and Antioxidant and Antimicrobial Activities of Rosemary (*Rosmarinus officinalis* L.).” Journal of Food Biochemistry 2023: 1–13. 10.1155/2023/6308773.

[emi470299-bib-0067] Santiesteban‐López, N. A. , J. A. Gómez‐Salazar , E. M. Santos , et al. 2022. “Natural Antimicrobials: A Clean Label Strategy to Improve the Shelf Life and Safety of Reformulated Meat Products.” Food 11: 2613. 10.3390/foods11172613. PMC9455744 36076798

[emi470299-bib-0068] Saucier, L. , C. Gendron , and C. Gariépy . 2000. “Shelf Life of Ground Poultry Meat Stored Under Modified Atmosphere.” Poultry Science 79: 1851–1856. 10.1093/ps/79.12.1851. 11194052

[emi470299-bib-0069] Schneider, G. , A. Steinbach , Á. Putics , Á. Solti‐Hodován , and T. Palkovics . 2023. “Potential of Essential Oils in the Control of *Listeria monocytogenes* .” Microorganisms 11: 1364. 10.3390/microorganisms11061364. 37374865 PMC10301788

[emi470299-bib-0070] Souza, E. L. , T. L. M. Stamford , E. O. Lima , and V. N. Trajano . 2007. “Effectiveness of *Origanum vulgare* L. Essential Oil to Inhibit the Growth of Food Spoiling Yeasts.” Food Control 18: 409–413. 10.1016/j.foodcont.2005.11.008.

[emi470299-bib-0071] Souza, E. L. D. , J. C. D. Barros , M. L. D. Conceição , N. J. Gomes Neto , and A. C. V. D. Costa . 2009. “Combined Application of *Origanum vulgare* L. Essential Oil and Acetic Acid for Controlling the Growth of Staphylococcus Aureus in Foods.” Brazilian Journal of Microbiology 40: 387–393. 10.1590/S1517-83822009000200032. 24031377 PMC3769715

[emi470299-bib-0072] Szumny, A. , A. Figiel , A. Gutiérrez‐Ortíz , and Á. A. Carbonell‐Barrachina . 2010. “Composition of Rosemary Essential Oil (*Rosmarinus officinalis*) as Affected by Drying Method.” Journal of Food Engineering 97: 253–260. 10.1016/j.jfoodeng.2009.10.019.

[emi470299-bib-0073] Takayama, C. , F. M. de‐Faria , A. C. A. De Almeida , et al. 2016. “Chemical Composition of *Rosmarinus officinalis* Essential Oil and Antioxidant Action Against Gastric Damage Induced by Absolute Ethanol in the Rat.” Asian Pacific Journal of Tropical Biomedicine 6: 677–681. 10.1016/j.apjtb.2015.09.027.

[emi470299-bib-0074] Thomas, F. , J.‐H. Hehemann , E. Rebuffet , M. Czjzek , and G. Michel . 2011. “Environmental and Gut Bacteroidetes: The Food Connection.” Frontiers in Microbiology 2: 93. 10.3389/fmicb.2011.00093. 21747801 PMC3129010

[emi470299-bib-0075] Vidaković Knežević, S. , S. Knežević , J. Vranešević , et al. 2023. “Effects of Selected Essential Oils on *Listeria monocytogenes* in Biofilms and in a Model Food System.” Foods 12: 1930. 10.3390/foods12101930. 37238748 PMC10217664

[emi470299-bib-0076] Vieira, M. , L. J. Bessa , M. R. Martins , et al. 2017. “Chemical Composition, Antibacterial, Antibiofilm and Synergistic Properties of Essential Oils From *Eucalyptus globulus* Labill. and Seven Mediterranean Aromatic Plants.” Chemistry & Biodiversity 14: e1700006. 10.1002/cbdv.201700006. 28281322

[emi470299-bib-0077] Wu‐Wu, J. W. F. , C. Guadamuz‐Mayorga , D. Oviedo‐Cerdas , and W. J. Zamora . 2023. “Antibiotic Resistance and Food Safety: Perspectives on New Technologies and Molecules for Microbial Control in the Food Industry.” Antibiotics 12: 550. 10.3390/antibiotics12030550. 36978417 PMC10044663

[emi470299-bib-0078] Yosr, Z. , C. Hnia , T. Rim , and B. Mohamed . 2013. “Changes in Essential Oil Composition and Phenolic Fraction in *Rosmarinus officinalis* L. Var. Typicus Batt. Organs During Growth and Incidence on the Antioxidant Activity.” Industrial Crops and Products 43: 412–419. 10.1016/j.indcrop.2012.07.044.

[emi470299-bib-0079] Yuan, W. , H. W. Lee , and H.‐G. Yuk . 2017. “Antimicrobial Efficacy of Cinnamomum Javanicum Plant Extract Against Listeria Monocytogenes and Its Application Potential With Smoked Salmon.” International Journal of Food Microbiology 260: 42–50. 10.1016/j.ijfoodmicro.2017.08.015. 28843123

